# Photochemically synthesized gold nanoparticles conjugated with Boswellic acid inhibit alpha synuclein aggregation and delay fibrillation kinetics

**DOI:** 10.1038/s41598-025-11107-6

**Published:** 2025-07-17

**Authors:** Masoumeh Gharb, Farima Mozafari, Payam Arghavani, Ali Akbar Saboury, Gholamhossein Riazi

**Affiliations:** 1 Institute of Biochemistry and Biophysics, University of Tehran, Tehran, 14176- 14335 Iran; 2 School of Chemical Engineering, University of Birmingham, Birmingham, B15 2TT UK

**Keywords:** α-synuclein, Boswellic acids, Gold nanoparticle, Protein fibrillation, Biochemistry, Biotechnology, Drug discovery

## Abstract

Neurodegenerative disorders are characterised by the gradual degeneration and death of neurons in distinct brain regions. In Parkinson’s disease (PD), a key pathological hallmark is the aggregation of misfolded α-synuclein (α-Syn) into neurotoxic fibrils. Although natural products such as Boswellic acids (BAs) from *Boswellia serrata* exhibit therapeutic potential, their clinical application is limited by poor pharmacokinetic properties. To address this, we investigated BAs conjugated to gold nanoparticles (GNPs) to enhance bioavailability and therapeutic potential. Both covalent and noncovalent β-Boswellic acid to GNPs (GNP-BA) conjugates were synthesized and evaluated for their effects on α-Syn fibrillation in vitro. The spherical GNPs (< 32 nm) were successfully characterized via high-resolution transmission electron microscopy (HR-TEM) and field emission scanning electron microscopy (FESEM). Conjugation of BA was confirmed by UV–Visible and Fourier-transform infrared (FTIR) spectroscopies. Thioflavin T (ThT) assay and atomic force microscopy (AFM) analyses demonstrated that noncovalently bound GNP-BAs significantly inhibited α-Syn fibril formation. Our findings reveal that both synthesis and conjugation strategies significantly influence the anti-aggregation behaviour of GNPs. Notably, photochemically synthesised GNP-BAs exhibited superior biofunctionality. This work introduces a dual-functional nanoplatform that enhances BA bioavailability while effectively inhibiting α-Syn aggregation, offering translational potential for real-world therapeutic development in PD and related synucleinopathies.

**Supplementary Information:**

The online version contains supplementary material available at 10.1038/s41598-025-11107-6.

## Introduction

The role of α-synuclein (α-Syn) in Parkinson’s disease (PD) has led to new understandings of the mechanisms behind this debilitating disorder^1^. The major pathological hallmarks of PD are degeneration of dopamine neurons in the substantia nigra and abnormal deposition of α-Syn into fibrillar Lewy bodies^2^. Although the physiological function of α-Syn remains unresolved, neuron degeneration in the affected brain regions is associated with the formation of α-Syn oligomers during its fibrillation process^3^. During this self-aggregation, α-Syn undergoes a structural transformation from intrinsically disordered monomers to form small cytotoxic soluble oligomers, which further assemble into protofibrils and eventually insoluble β-sheet rich fibrils^4,5^. These intermediate oligomers have been identified as highly damaging to cellular function, and are considered the most neurotoxic forms of α-Syn^5,6^. The toxicity of certain α-Syn oligomers is attributed to specific structural features, which lead to a range of detrimental effects including disrupting cell membranes especially mitochondrial, and synaptic impairment^7,8^. As people age, certain small protein fragments and peptides become more prone to aggregation, which can lead to the formation of harmful species. This process is especially apparent in progressive amyloidosis disorders such as PD. The increasing incidence of such diseases in an aging population has led to significant societal costs^9,10^. Therefore, the identification of compounds capable of inhibiting or delaying the aggregation mechanisms associated with neurotoxicity could be a promising approach to preventing or treating PD.

Natural products, with their complex molecular frameworks, offer a diverse array of chemical species for medicinal chemists to explore in discovering chemical probes and drugs. For a long time, these natural compounds have been a valuable resource in drug development and treating various diseases. Recently, certain natural products, such as Boswellic acid, have gained significant attention for their potential in treating neurodegenerative complications^10,11^. β-Boswellic acid (BA), a pentacyclic triterpene derived from frankincense resin obtained from the Boswellia serrata tree, has been studied extensively. BA has demonstrated potent efficacy in treating various neurological diseases^12,13^. However, bioavailability has been a major hurdle in translating the preclinical potential of BA into therapeutic effects^14^. Various approaches have been employed to improve BA’s bioavailability, including lecithin formulation, standardized meal administration, and oral co-administration, which have shown enhanced bioavailability and therapeutic effects^15^. Notably, novel drug delivery systems like nanoparticles (NPs) introducing chemically active surfaces and high surface-to-volume ratios have been proven effective in increasing the bioavailability and pharmacokinetic properties of BA^16^.

Gold nanoparticles (GNPs) are considered one of the most biocompatible nanocarriers for drug delivery due to their physicochemical properties, ease of synthesis in various sizes and shapes, minimal toxicity, and excellent penetration into the blood-brain barrier. Due to their significant surface area, GNPs can carry high drug payloads and enhance drug efficacy in a controlled manner while minimizing the potential for adverse effects^16,17^.

Consequently, there has been growing interest in combining BA with GNPs, leveraging the unique physical, chemical, and biological properties of the resulting hybrid materials. In addition to the valuable pharmaceutical effects of BA, conjugation of GNPs with BA is a functionalization approach of NPs in nature, a promising strategy to achieve significant biocompatibility and improved colloidal stability, as well as minimizing unwanted biological responses such as the challenge protein corona formation^18,19^. The inhibition of α-Syn fibrillation using metal NPs has been studied broadly^20,21^ suggesting that GNPs potentially may interact and disassemble amyloid fibrils. Therefore, in this investigation, we aim to examine the inhibitory effect of GNP-BA on the fibrillation of α-Syn in vitro.

## Materials and methods

### Materials

Tetrachloroauric acid (HAuCl4), 1-ethyl-3-(3dime-thylaminopropyl) carbodiimide (EDC), N-hydroxy succinic-mide (NHS), 2-(N-111Morpholino) ethane sulfonic acid (MES), trisodium citrate, dimethyl sulfoxide (DMSO), thioflavin T (ThT), isopropyl-D-1-thiogalactopyranoside (IPTG) from Sigma-Aldrich (Munich, Germany). PT7-7 α-Syn WT plasmid containing the α-Syn gene was obtained from Addgene. The HiTrap Q FF anion exchange chromatography column was from GE Healthcare. Tween 20, and Triton X-100 (TX-100) were purchased from Merck (Darmstadt, Germany). Boswellic acid was a gift from Kondor Pharma Inc. (Canada). Deionized water was used for making all solutions.

### Preparation of GNP-BAs using the chemical method

GNPs were synthesized using the Turkevich method (C-G). Trisodium citrate (1%) was added to boiling chloroauric acid (0.8 mM), changing its color to red in 15 min. After the solution cooled, BA was conjugated to the GNPs using EDC/NHS chemistry. The GNPs were first mixed with Tween 20 and cysteamine in an MES buffer. EDC (2 µM) was then added to BA, followed by NHS (4 µM), and the mixture was added to the cysteamine-coated GNPs. This reaction was gently shaken for 48 h. The GNPs were then centrifuged at 10,000 × g for 30 min to remove excess reagents. The final GNP-BA conjugates (C-G-BA) were stored at 4 °C^22^.

### Preparation of GNP-BAs using the physical method

GNPs were synthesized by subjecting a solution of HAuCl₄ and TX-100 to UV irradiation for 10 min (U-G). For conjugation, the GNPs were mixed with BA (5 mM) in DMSO and incubated for 48 h. After incubation, the mixture was centrifuged to remove excess BA, and the GNP-BA conjugates (U-G-BA) were stored at 4 °C^22^.

### Physicochemical properties of GNP and GNP-BA

The spectra of GNPs and GNP-BA were measured using a UV − visible spectrophotometer (Carry 100 Bio Varian) over a wavelength range of 200–800 nm. A Fourier transform infrared spectrometer (Irprestige-21, Shimadzu) was employed to assess the chemical interactions of different functional groups, with spectra recorded in the range of 4000 to 400 cm⁻¹ at a resolution of 4 cm⁻¹. The particle size, size distribution, and surface charge were analyzed using dynamic light scattering (DLS) and ζ potential measurements (Brookhaven ZetaPlus ζ Potential Analyzer). The surface morphology, size distribution, and crystallinity of the GNPs were examined using a high-resolution transmission electron microscope (FEI Tecnai G2 F20 SuperTwin) operating at an accelerating voltage of 200 kV. A drop of each sonicated and monodispersed GNP sample in deionized water was placed on a transparent carbon-coated copper grid. Elemental composition analysis was performed on a field emission scanning electron microscope (Zeiss 436 Sigma VP) with a carbon-coated copper tape grid. Images were analyzed using ImageJ software.

### Expression and purification of αSyn

α-Syn was expressed in *Escherichia coli* BL21(DE3) cells transfected with pT7-7 α-Syn wild type (WT) plasmid, following the method of Hoyer et al. with some modifications^23^. Briefly, the transfected *E. coli* cells were grown overnight in Luria broth (LB) containing 100 µg/mL ampicillin. The next day, the pre-culture was used to inoculate fresh LB medium. When the culture reached an OD_600_ of 0.6, α-Syn expression was induced by adding 1 mM IPTG, then incubated at 37 °C while shaking at 180 rpm for 4 h. Cells were then harvested by centrifugation at 6000 rpm for 5 min at 4 °C to obtain the cell pellet. The pellet was resuspended in lysis buffer (20 mM Tris base pH 8.0, 1 mM EDTA, and 1 mM PMSF), and the cells were lysed by sonication (50 W, 10 s on and 10 s off). The lysed cells were then incubated in boiling water for 20 min, followed by centrifugation at 18,000*g* for 30 min at 4 °C. The supernatant was collected, and ammonium sulfate was slowly added to a final concentration of 0.36 g/mL. After stirring for 30 min at 4 °C, the mixture was centrifuged at 18,000*g* for 20 min at 4 °C. The resulting pellet was resuspended in 20 mM Tris buffer (pH 8.0) and loaded onto a HiTrap Q FF anion exchange chromatography column. α-Syn was eluted using 300 mM NaCl, and its purity (≥ 95%) was confirmed by SDS-PAGE. The purified α-Syn was dialyzed against 20 mM Tris base buffer (pH 7.5), and its concentration was determined by measuring absorbance at 275 nm (ε_275_ = 5600 M^− 1^.cm^− 1^). Notably, the purified α-Syn used in our studies didn’t contain any additional motifs, such as a His-tag, that could interact with the metal ions used in our experiments^23^.

### Aggregation of αSyn

To study the effect of BA, GNP, and GNP-BA on α-Syn fibril formation, 90 µM of α-Syn in 20 mM Tris buffer (pH 7.5) was incubated with each sample at 37 °C while stirring at 1000 rpm for 60 h. As a control, α-Syn was incubated in Tris buffer alone. Kinetic studies were conducted during the incubation period, followed by characterization of the fibrils after the completion of fibril formation^24^.

### Thioflavin (ThT) fluorescence assay

A steady-state ThT kinetic assay was conducted to study α-Syn aggregation, with excitation at 440 nm and emission at 482 nm, using a Cary Eclipse fluorescence spectrophotometer (Varian). Cuvette wells were filled with a final sample volume of 200 µL, containing 90 µM α-Syn and 20 µM ThT.

### Circular dichroism spectropolarimetry (CD)

Far-UV circular dichroism (CD) spectra were recorded using an AVIV 215 spectropolarimeter to analyze the secondary structural changes of α-Syn at the end of fibril formation. The measurements were conducted at room temperature in the presence of C-G-BA and U-G-BA, covering the wavelength range of 190–260 nm.

### Atomic force microscopy (AFM)

α-Syn fibrils formed in the presence and absence of C-G-BA and U-G-BA were analyzed by AFM. Images were obtained in semi-contact mode using an AFM (NTEGRA, NT-MDT, Russia) and processed with Nova software (version 1.26.0.1443).

## Result and discussion

In this study, we have meticulously synthesized GNPs through two distinct methods. The physicochemical attributes of the synthesis process play a pivotal role in the size, morphology, and functional properties of these NPs^25,26^. Subsequently, we employed BA, known for its therapeutic potential in addressing neurodegenerative disorders, as a conjugation agent with GNPs. Our objective was to enhance the potential GNP efficacy in neurodegenerative treatment alongside improving their stability, biocompatibility, and bioavailability. The results presented in this article explore the intricacies of this innovative strategy.

**Fig. 1:**
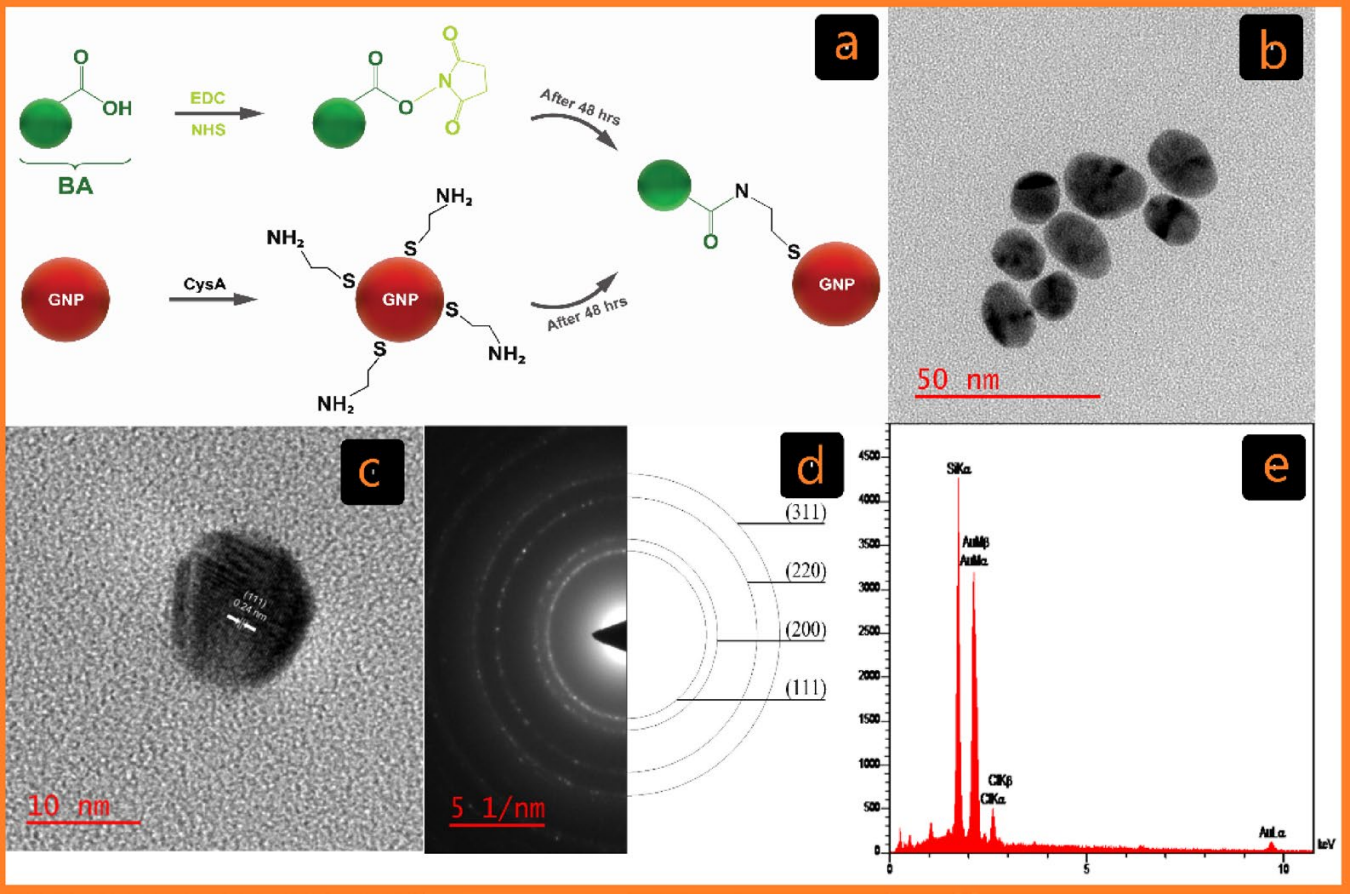
Characterization of C-G. (**a**) Schematic of the cross-linking GNP reactions with BA Functionalized by EDC/NHS in covalent conjugation (C-G-BA). (**b**) TEM image displaying C-G and its associated size distribution. (**c**) HR-TEM image of C-G. (**d**) Selected area electron diffraction (SAED) pattern. (**e**) EDX analysis of C-G via FE-SEM.

The Turkevich method, the most common chemical method for the synthesis of GNPs in biological applications, involves the reduction of tetrachloroauric acid (HAuCl4) with trisodium citrate to produce GNPs (C-G)^27^. Over the past two decades, the development of covalent conjugation techniques has significantly advanced research in biomedicine, materials science, and nanotechnology^28^. In this study, GNPs were conjugated with BA using cysteamine (CysA) as a linker and EDC/NHS as a cross-linker to establish covalent bonds (Fig. 1a)^28^.

Another effective method for producing GNPs (U-G) is photochemical synthesis^29^. While each method for synthesizing GNPs has its advantages and disadvantages, chemical methods are efficient but often involve toxic reducing agents, which pose biological risks. In contrast, photochemical synthesis is a non-toxic, eco-friendly alternative that is increasingly recognized for producing GNPs suitable for various biological applications. This method utilizes UV irradiation in the presence of Triton X-100 micelles, which play a crucial role in stabilizing the GNPs and influencing their morphology (U-G)^30^.

**Fig. 2:**
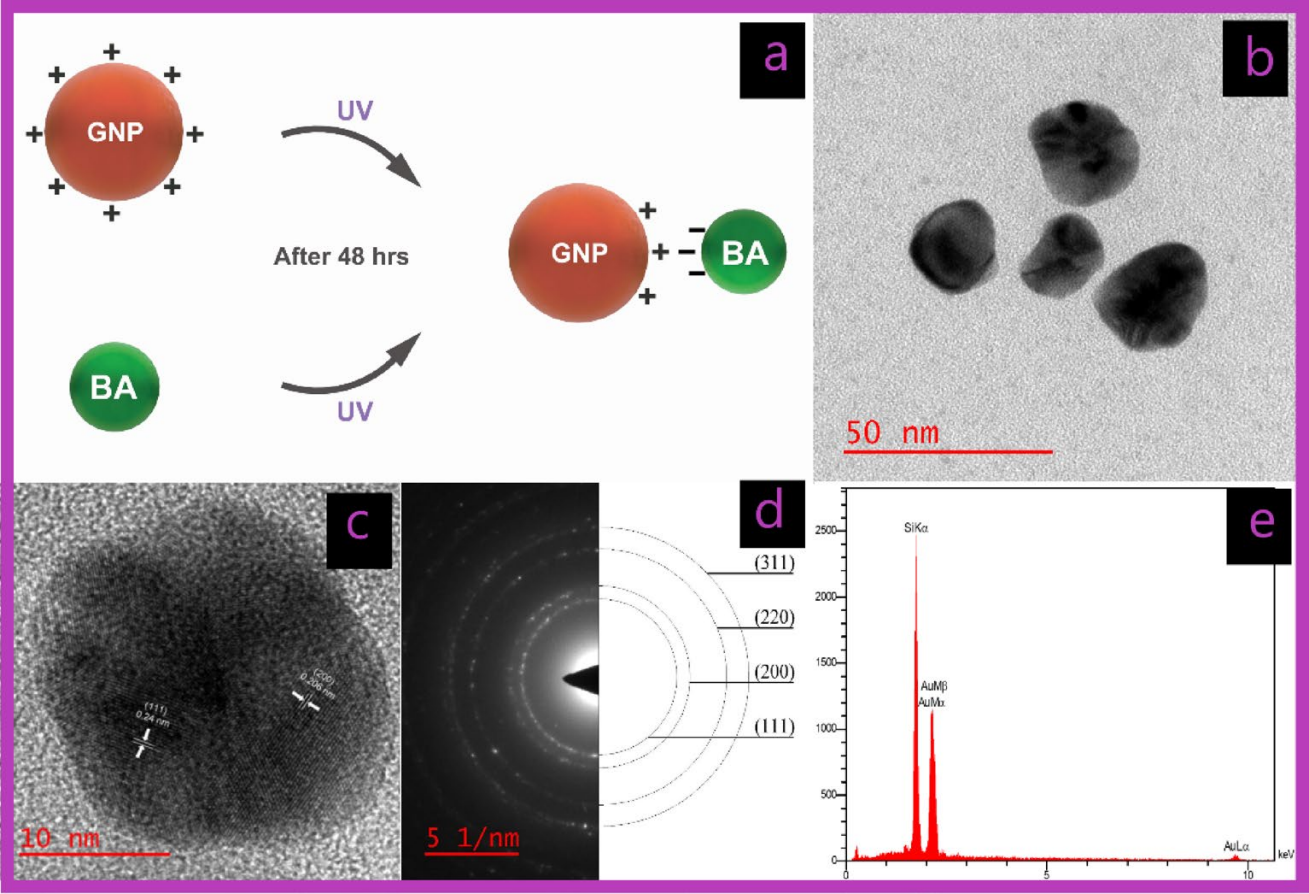
Characterization of U-G. (**a**) Schematic of the noncovalent conjugation of GNPs with BA (U-G-BA). (b) TEM image showing U-G and its corresponding size distribution. (c) HR-TEM image of U-G. (d) Selected area electron diffraction (SAED) pattern. (e) EDS analysis of U-G via FE-SEM.

Covalent interactions lead to the creation of new molecules with distinct properties and are generally stronger than noncovalent interactions^31,32^. However, for smaller drug molecules bound to NP surfaces via covalent bonds, these bonds must be broken for the drug release to function effectively. Therefore, for targeted therapy, using non-covalent interactions to create nanoconjugates may be more effective. Such nanoconjugates are stable enough to reach the intended site in the body after administration^33^. This approach ensures both stability and functionality, thereby enhancing drug delivery efficacy. Accordingly, in this study, the GNPs were also coated with BA using noncovalent electrostatic interactions (U-G-BA) (Fig. 2a).

### Fabrication and characteristics of C-G-BA and U-G-BA

The chemical and physical properties of the synthesized GNPs were characterized using a combination of UV-Vis spectroscopy, DLS, ζ potential, FT-IR, and HR-TEM. The formation of GNPs was further confirmed by EDX analysis via FE-SEM. Visual inspection was used to track the formation of C-G-BA and U-G-BA, with a change from light-yellow color to wine red confirming the synthesis. This color transformation corresponds to the surface plasmon resonance (SPR) feature of the produced GNPs^34^. After three rounds of centrifugation and washing with deionized water, the GNP samples were examined with UV–Vis spectroscopy. SPR band of C-G was observed at 522 nm, and after conjugation with BA (C-G-BA), a redshift to 524 nm was noted. To optimize BA concentration for conjugation with C-G, various concentrations (1, 5, 10, 15, 20, and 25) were tested, with the results indicating that a BA concentration of 5 was the most effective. Similarly, the surface plasmon band of U-G-BA exhibited a slight redshift, shifting from 530 nm to 536 nm compared to U-G (Figure S1a). This redshift is due to a change in the dielectric environment of the GNPs, confirming the effective conjugation of BA to GNPs in both cases.

HR-TEM analyses were conducted to evaluate the size and morphology of the GNPs. The images depicted in Figs. 1b and 2b show spherical-shaped GNPs with an average diameter of 16.48 ± 2.13 and 31.23 ± 6.21 for C-G and U-G respectively. The selected area electron diffraction (SAED) pattern confirmed the crystalline nature of the GNPs, revealing bright circular rings (Figs. 1d and 2d). These rings corresponded to reflections from the standard Bragg planes (111), (200), (220), and (311), indicating that the GNPs had a cubic crystal structure (Figs. 1c and 2c)^35^. Energy-dispersive X-ray spectroscopy (EDX) revealed strong peaks at 2.15 keV, typical of metallic gold nanocrystallites, due to surface plasmon resonance (Figs. 1e and 2e)^36^. We have previously demonstrated and characterized the successful synthesis of leveraged GNPs^22^. Functionalization and BA conjugation were also confirmed by using DLA, ζ potential, and FTIR. Additionally, these NPs are negatively charged and exhibit significant colloidal stability (Table 1).

**Table 1:** Surface charge and size of synthesized gnps.

Nanoparticles	ξ potential (mV)*	particle size (nm)	
		DLS (nm)*	HR-TEM (nm)
C-G	−25.59 ± 2.02	23.4	16.48 ± 2.13
C-G-BA	−15.74 ± 0.76	27.4	–
U-G	+ 8.16 ± 0.86	48.2	31.23 ± 6.21
U-G-BA	−22.56 ± 1.51	55.5	–

*Adapted from our previous study^22^.

NPs hydrophobicity, size, and surface charge are physicochemical properties that are known to affect protein aggregation^37,38^. It is well documented that surface modifications of NPs can alter protein aggregation pathways, either slowing down or accelerating aggregation, and potentially even reversing preformed aggregates^39,40^. For instance, modifying the iron oxide NPs’ surface with leucine made them more hydrophobic and significantly blocked mTTR’s aggregation and fibrillation pathways^41^. Additionally, these modifications have the potential to sequester misfolded states or even correct their conformation. GNPs have been extensively studied for their interactions with amyloidogenic proteins, which are associated with neurodegenerative diseases^39^. Specifically, GNPs-BA may interact with α-Syn and lead to inhibiting its aggregation and fibrillation processes.

**Fig. 3:**
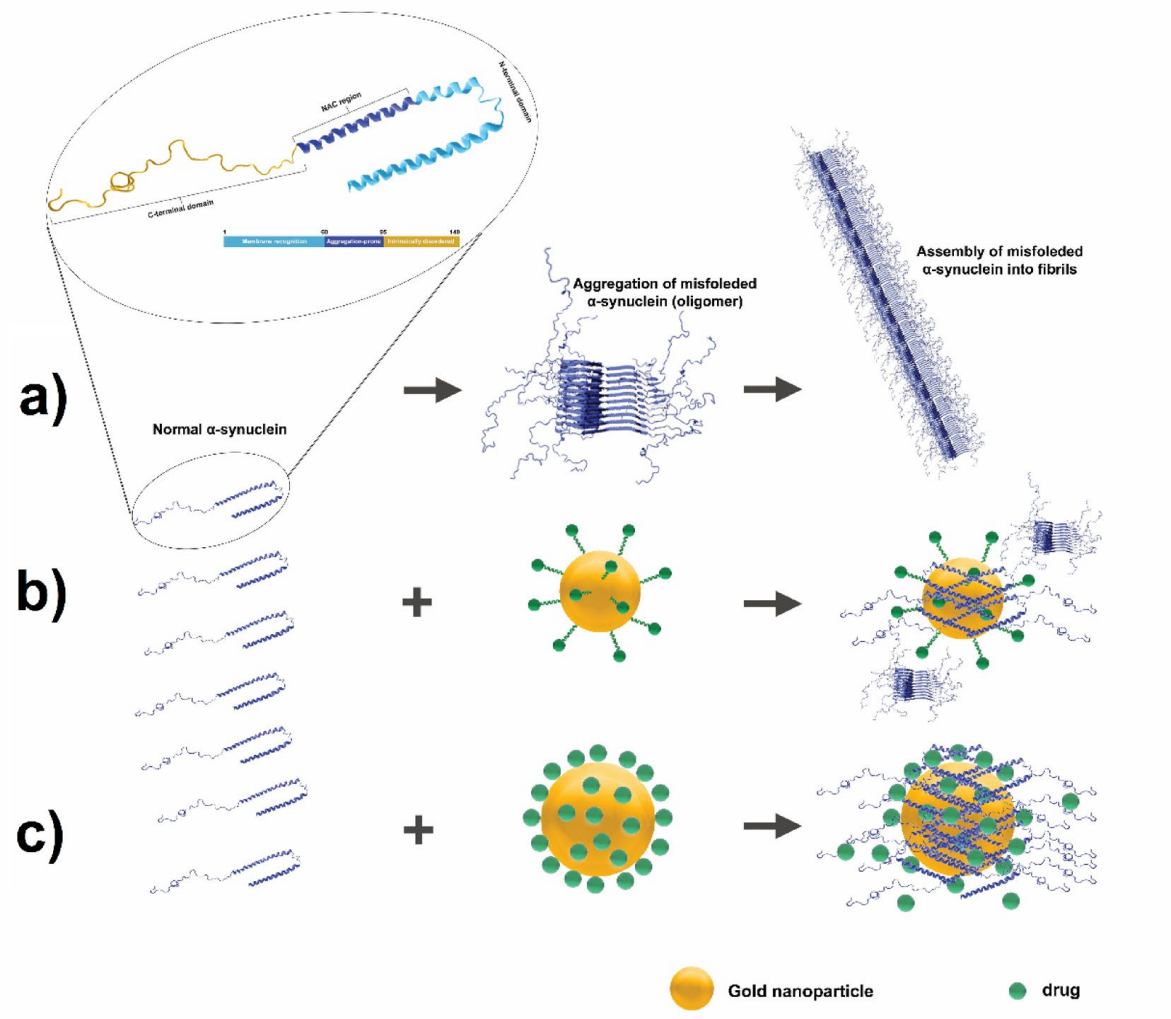
Mechanistic illustration of α-Syn aggregation and its inhibition by GNPs conjugated with BA. (**a**) Native α-Syn undergoes misfolding, leading to the formation of oligomers that aggregate into fibrils. (**b**) Interaction of α-Syn with C-G-BA partially inhibits fibrillation. (**c**) Enhanced inhibition of α-Syn aggregation using U-G-BA, effectively preventing fibril formation.

### The presence of C-G-BA & U-G-BA NPs alters the aggregation kinetics of α-Syn

The kinetics of α-Syn fibril formation were studied in the absence and presence of increasing concentrations of bare GNPs, GNP-BA, and pure BA (Fig. 3). This process was monitored by tracking the characteristic increase in Thioflavin T (ThT) fluorescence intensity^42,43^. Typically, amyloid aggregation kinetics are characterized by a sigmoidal curve, which includes a lag phase, a growth phase, and a final equilibrium phase^44^. The impact of GNP-BA on both the nucleation (lag time) and elongation (exponential phase) processes was quantified using kinetic parameters derived from data fitting. Regardless of the specific amyloid protein being studied, experimental data are generally fitted to this sigmoidal model^45^ (Eq. 1):

1 $$\:F=\:\frac{{F}_{final}-\:{F}_{0}}{1+exp(-{k}_{app}(t-\:{t}_{1/2}\left)\right)}$$

In this equation, F represents the fluorescence intensity at time *t*, *F*_*final*_ denotes the maximum fluorescence intensity, $$\:{t}_{\raisebox{1ex}{$1$}\!\left/\:\!\raisebox{-1ex}{$2$}\right.}$$ is the time required to reach half of the maximum fluorescence intensity (corresponding to the midpoint between nuclei formation and fibril growth), and *k*_*app*_ stands for the apparent first-order aggregation constant. The lag time is defined as the point where the tangent at the maximum fibrillation rate intersects the abscissa, as given by^46^ (Eq. 2):

2 $$\:{t}_{lag}=\:{t}_{1/2}-\:{2k}^{-1}$$.

**Fig. 4:**
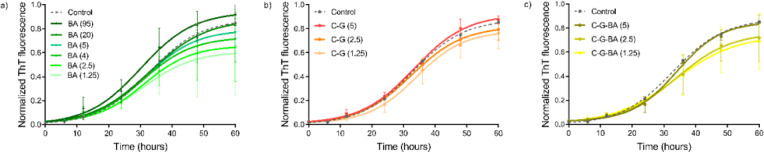
Aggregation profile of α-Syn in the presence of chemically prepared GNPs, measured by ThT assay. Effect of the (**a**) BA concentration (1.25 − 95 µM), (**b**) C-G concentration (1.25 − 5 µM), and (**c**) C-G-BA concentration (1.25 − 5 µM).

In several studies, BA has been utilized to treat neurodegenerative diseases at concentrations ranging from 1 to 100 µM^47,48^. In our experiments, we investigated concentrations from 1.25 to 95 µM. Consistent with previous reports, we found that concentrations up to 20 µM inhibit aggregation (Table 2; Fig. 4). However, at higher concentrations, BA appears to promote fibril formation by decreasing the lag time and increasing the plateau phase magnitude (Fig. 4a), thereby accelerating the formation of fibril seeds and shortening the lag time. This behavior indicates that BA not only affects nucleation processes but also plays a role in the availability of monomers for subsequent fibril elongation and growth^49^.

We observed that negatively charged C-G (citrate-capped), slightly accelerated the aggregation of α-Syn at concentrations ranging from 1.25 to 5 µM, as evidenced by a slight reduction in the lag time and a small increase in plateau intensity (Table 2; Fig. 4b). These findings are consistent with previous research, which also reported that NP surface charge and concentration can modulate protein aggregation pathways. This underscores the dual role of NP in either inhibiting or promoting fibril formation, depending on their physicochemical properties and the environment^20^. It is well known that fibrillation is a nucleation-dependent mechanism in which nucleation is triggered by external factors, including NP interactions. NPs act as catalysts that facilitate the assembly of peptides into stable fibril aggregates by increasing the local concentration of those peptides and promoting the formation of nucleation sites^50^.

α-Syn consists of three structural domains: the N-terminal domain (residues 1–60), the central non-amyloid component (NAC) domain (residues 61–95), and the C-terminal domain (residues 96–140). The N-terminal region is predominantly positively charged due to the presence of 11 lysine residues, whereas the C-terminal domain is negatively charged^51^. C-G appears to interact with the N-terminal region of α-Syn, potentially exposing the NAC region. This exposure may result in higher local concentrations of the NAC domain, thereby facilitating aggregation^20^.

When studying C-G-BA at concentrations ranging from 1.25 to 5 µM, fibril formation declined by increased lag time compared to both BA and C-G alone, indicating strong inhibition of nucleation. The rate of fibril formation also decreased with increasing concentrations of C-G-BA (Table 2; Fig. 4c). This suggests that the presence of BA slows down the growth kinetics of fibril formation. The extended lag time suggests that C-G-BA raises the energy barrier for nucleation more effectively than BA or C-G alone. The interactions between the C-G-BA and α-Syn may prevent the formation of fibril nuclei by competing with monomer-monomer interactions. The plateau phase intensity is lower than that of C-G, indicating reduced fibril formation (Fig. 7). These results suggest that C-G-BA limits monomer addition to fibril ends^20^.

**Fig. 5:**
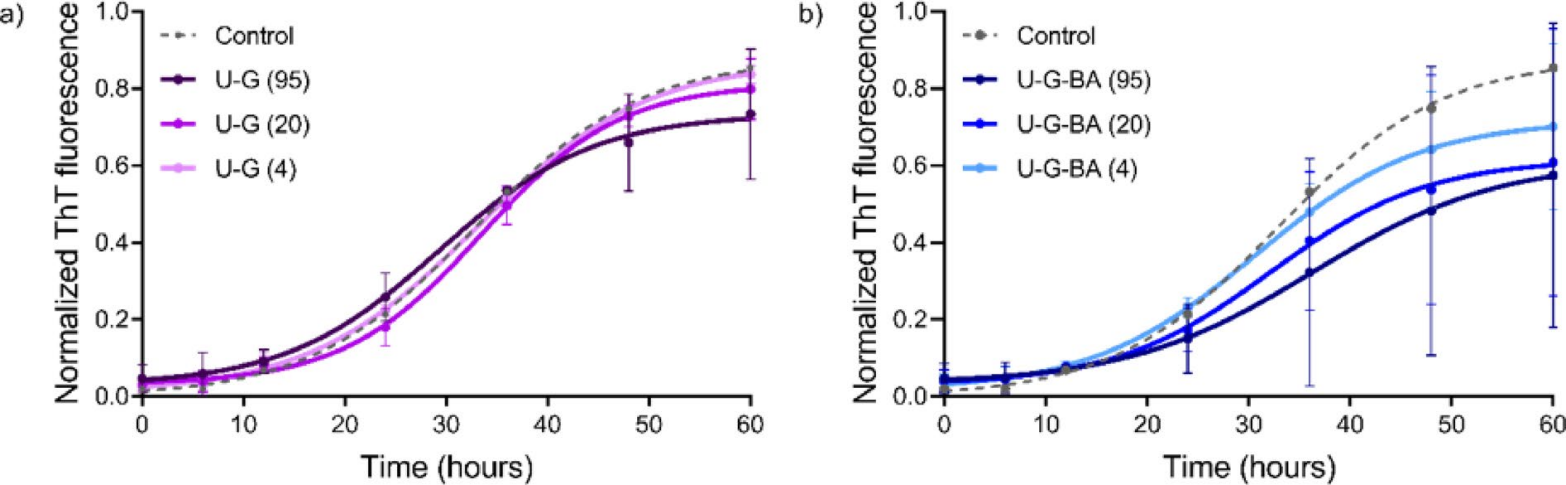
Aggregation profile of α-Syn in the presence of physically prepared GNPs, measured by ThT assay. Effect of the (**a**) U-G concentration (4–95 µM), and (**b**) U-G-BA concentration (4 − 95 µM).

We observed that positively charged U-G Triton (X-100 capped), at concentrations ranging from 4 to 95 µM, decreased the aggregation of α-Syn, as indicated by an increase in the lag time, shortening the growth phase, and reducing the plateau phase intensity. This suggests that U-G also partially inhibits elongation by interfering with monomer addition to fibril ends. The positively charged surface of U-G likely binds to the negatively charged C-terminal region of α-Syn, preventing nucleation and fibril elongation (Table 2; Fig. 5a). At higher concentrations, the lag time decreased, but the plateau phase intensity significantly decreased, suggesting that U-G exerts a concentration-dependent effect. The binding of a large number of monomers to U-G at higher concentrations may result in a depletion of free monomers, limiting their availability for elongation and reducing the overall fibril content. Previous studies have shown that positively charged NPs can bind to the C-terminus of α-Syn. The positively charged U-G are expected to adsorb/capture a large number of α-syn monomers, making them promising candidates to prevent or delay the fibrillation process^52^.

The U-G-BAs, studied at concentrations ranging from 4 to 95 µM, significantly extended the lag phase and impacted the elongation phase (Table 2; Fig. 5b), and reduced plateau intensity compared to both U-G and BA. This inhibitory effect of U-G-BA was concentration-dependent, with the most pronounced increase in the lag phase at the highest concentration of 95 µM. The interactions between the U-G-BA surface and α-Syn monomers and/or oligomers might create unfavorable conditions for fibril growth by obstructing binding sites for the addition of new monomers^53^. U-G likely binds to the C-terminal region of α-Syn, while BA interacts with other aggregation-prone regions, such as the NAC domain, stabilizing α-Syn in its monomeric form and delaying the formation of fibril nuclei. The significant reduction in the plateau intensity suggests that U-G-BA inhibits overall fibrillation by limiting both nucleation and elongation.

The larger diameter of U-G-BA provides an increased surface area for stronger interactions with α-Syn, enhancing its ability to block nucleation and elongation. Its stronger electrostatic interactions with the negatively charged C-terminal region of α-Syn allow more effective sequestration of monomers and oligomers compared to C-G-BA. Additionally, its lower surface passivation ensures greater binding efficiency, making U-G-BA a more effective inhibitor of fibrillation. Moreover, the behavior of U-G-BA in inhibiting fibril formation is reminiscent of chaperone-like activity. Chaperone proteins assist in the proper folding of other proteins and prevent misfolding and aggregation. U-G-BA, through its interactions and enhanced solubility, mimics this chaperone-like function by stabilizing α-Syn in its non-fibrillar form and preventing the progression of aggregation^54^.

**Table 2:** Kinetic parameters of α-Syn fibril formation in the presence of various concentrations of gnps, BA, and GNP-BA collected from the best fit.

	k_app_ (h^− 1^)	t_1/2_ (h)	t_lag_ (h)
BA (0.05)	0.10444$\:\pm\:$0.015	32.86$\:\pm\:$1.088	13.71025$\:\pm\:$2.706
BA (0.2)	0.10782$\:\pm\:$0.020	31.75$\:\pm\:$0.991	13.20057$\:\pm\:$2.463
BA (1)	0.10888$\:\pm\:$0.026	31.07$\:\pm\:$1.219	12.70115$\:\pm\:$2.391
BA (4)	0.10894$\:\pm\:$0.015	31.11$\:\pm\:$1.392	12.75127$\:\pm\:$2.387
BA (20)	0.09668$\:\pm\:$0.020	30.08$\:\pm\:$1.219	9.393198$\:\pm\:$3.370
BA (95)	0.11582$\:\pm\:$0.038	27.32$\:\pm\:$0.993	10.05183$\:\pm\:$1.973
C-G (0.05)	0.11566$\:\pm\:$0.026	34.06$\:\pm\:$1.088	16.76794$\:\pm\:$1.973
C-G (0.2)	0.10572$\:\pm\:$0.154	33.28$\:\pm\:$0.991	14.3621$\:\pm\:$1.982
C-G (1)	0.10384$\:\pm\:$0.020	32.53$\:\pm\:$1.219	13.2696$\:\pm\:$2.611
C-G-BA (0.05)	0.0903$\:\pm\:$0.020	33.52$\:\pm\:$1.088	11.37161$\:\pm\:$2.752
C-G-BA (0.2)	0.10116$\:\pm\:$0.026	34.03$\:\pm\:$0.991	14.25934$\:\pm\:$4.058
C-G-BA (1)	0.12282 + 0.038	34.03$\:\pm\:$1.219	17.74601$\:\pm\:$2.967
U-G (4)	0.10422$\:\pm\:$0.023	33.05$\:\pm\:$1.392	13.85983$\:\pm\:$1.623
U-G (20)	0.12736$\:\pm\:$0.022	33.52$\:\pm\:$1.013	17.81648$\:\pm\:$2.723
U-G (95)	0.1143$\:\pm\:$0.022	29.4$\:\pm\:$0.988	11.90219$\:\pm\:$1.428
U-G-BA (4)	0.11272$\:\pm\:$0.021	30.65$\:\pm\:$1.032	12.90692$\:\pm\:$2.150
U-G-BA (20)	0.10644$\:\pm\:$0.017	32.18$\:\pm\:$1.234	13.39007$\:\pm\:$2.559
U-G-BA (95)	0.09456$\:\pm\:$0.013	36.28$\:\pm\:$1.876	15.12941$\:\pm\:$3.582

Our results suggest that the observed changes in the lag time primarily reflect modulation of nucleation, as the strong inhibition by U-G-BA and C-G-BA indicates that these systems stabilize α-Syn by interacting with aggregation-prone domains, such as the N-terminal or NAC regions. These interactions reduce the local concentration of free α-Syn monomers, and the primary effect is on early-stage aggregates, preventing the initiation of new nuclei^55^.

To address this issue, ThT kinetics data were confirmed by AFM and far-UV circular dichroism (CD) analyses, which provide valuable information on the morphology and extent of α-syn fibrils. These findings provide insight into the interactions between GNP-BA and α-Syn, suggesting the possibility of targeting related amyloid formations beyond central nervous system aggregation. Building on evidence from studies where GNPs modulate microbial amyloid formation and biofilm development through gut microbiota remodeling, we hypothesize that GNP-BA may similarly target microbial amyloids associated with gut biofilms. Given its demonstrated inhibitory effects on α-Syn aggregation through ThT fluorescence and AFM imaging, GNP-BA is likely capable of disrupting β-sheet-rich amyloids through hydrophobic and electrostatic interactions. This potential suggests that GNP-BA could play a role in managing amyloid-related processes within the gut, which are hypothesized to contribute to PD progression via the gut-brain axis^56–58^.

**Fig. 6:**
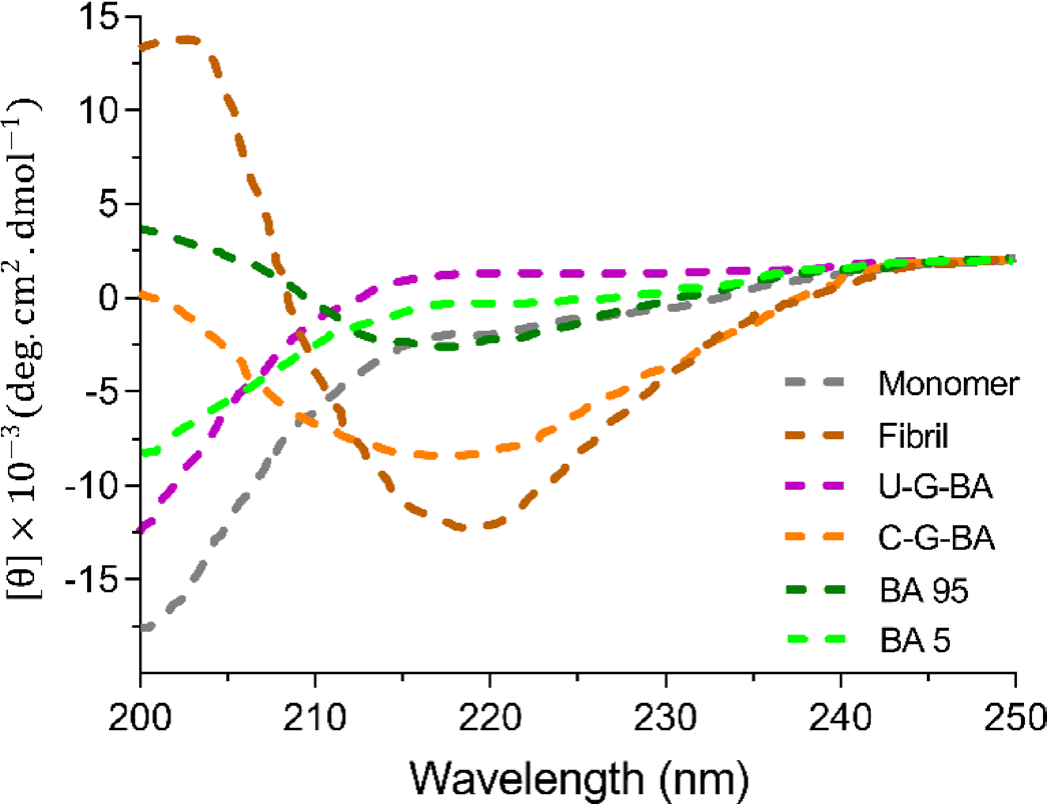
Far-UV CD spectra showing α-Syn monomers and fibrils, comparing samples formed in the absence (monomer) versus the presence of C-G-BA (5 µM) and U-G-BA (95 µM).

The Far-UV CD spectra of α-Syn (Fig. 6) resemble the classical spectrum of a disordered protein, while the β-sheet structures of fibrils are characterized by a negative minimum around 218 nm and a positive peak at 202 nm respectively^59^. The Far-UV CD signature became more complex in the presence of GNP-BA, suggesting alterations in the α-syn’s secondary structure. Conversely, C-G-BA exhibited a weak band at 218 nm, indicative of reduced fibril formation and the potential emergence of alternative fibril morphologies. U-G-BA effectively preserved α-Syn’s native secondary structure and inhibited fibril formation, as reflected by the absence of notable spectral changes. Additionally, the Far-UV CD spectrum of α-Syn in the presence of BA at 5 µM suggested the protein predominantly remained monomeric, whereas, at 95 µM, a transition to aggregated states was observed, characterized by a negative minimum at 216 nm.

**Fig. 7:**
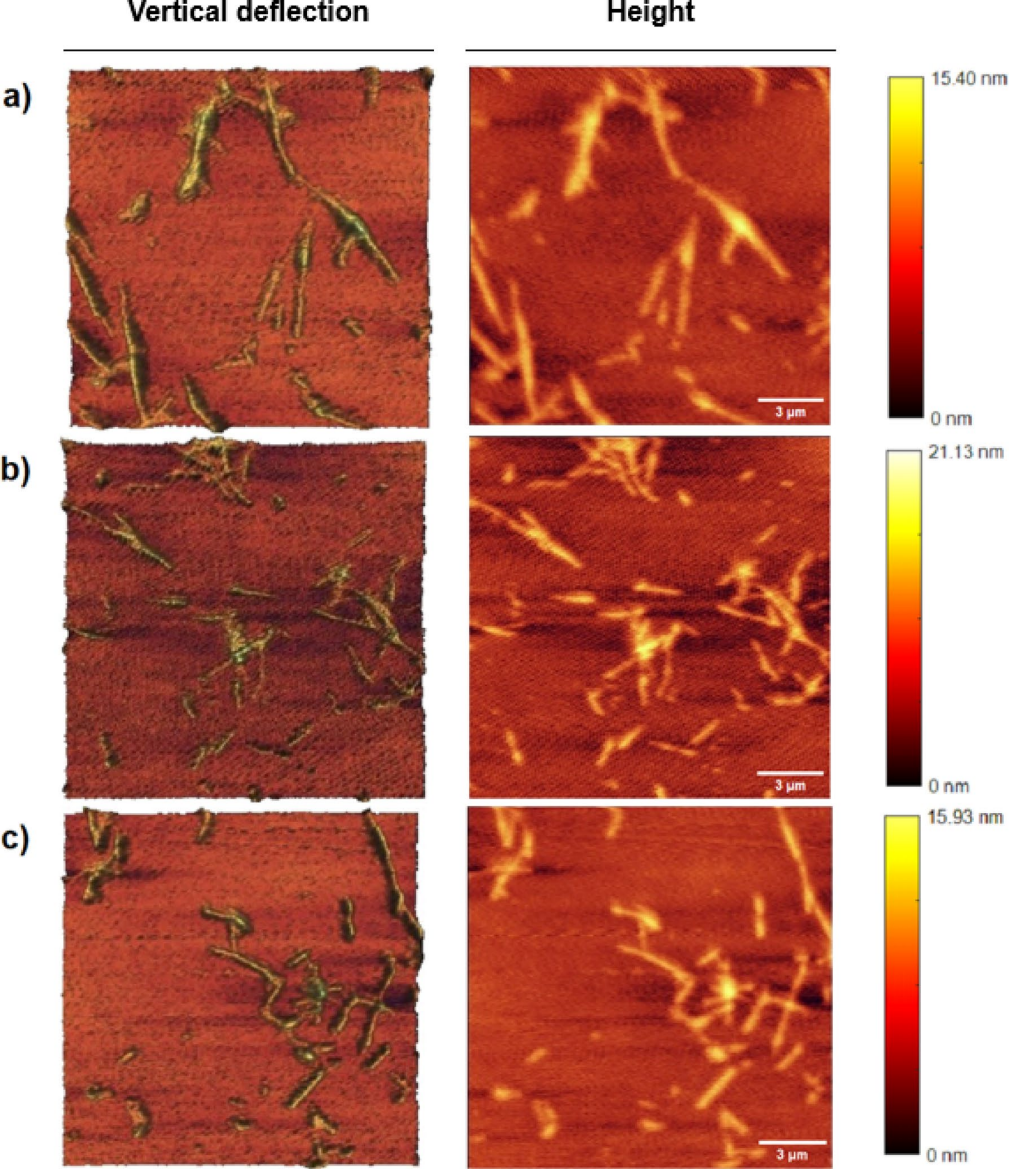
AFM images of (**a**) α-Syn fibril, (**b**) α-Syn fibrils formed in the presence of 5 µM concentration of C-G-BA, and (**c**) α-Syn fibrils formed in the presence of 95 µM concentration of U-G-BA.

AFM has been utilized as a suitable tool to determine possible rearrangements between the protofibrils producing a fibrillar polymorphism in the mature fibril. AFM in the control sample, which was untreated, the fibrils exhibited an elongated and intertwined morphology with heights reaching up to 15.40 nm and average lengths of 1.2 μm. These fibrils were uniformly structured, indicating a stable morphology with minimal height variation (Fig. 7a). Treatment with C-G-BA led to more complex fibril structures, with heights extending up to 21.13 nm and average lengths of 0.54 µm.

The AFM images revealed a heterogeneous arrangement of fibrils, including straight and curved forms with varying densities among treated samples. This suggests that C-G-BA treatment induced significant structural changes, resulting in a more pronounced and varied fibril morphology compared to the control. The increased height range and diverse structural features indicated a more extensive aggregation pattern (Fig. 7b). In contrast, U-G-BA treatment resulted in fibrils with heights up to 12.55 nm and average lengths of 0.39 μm. The AFM images showed elongated, thread-like structures with less pronounced height variation and a more dispersed distribution. This pattern suggests that U-G-BA treatment is associated with reduced aggregation compared to C-G-BA, as indicated by the smaller and fewer aggregates. The less pronounced height variation in the U-G-BA-treated fibrils further supports the notion that this treatment leads to a less structured fibril network (Fig. 7c)^60^. U-G-BA largely determines the polymorphism of fibrils^61^.

Among several therapeutic approaches explored for Alzheimer’s and PD in recent years, efforts have been made to use interfering molecules as fibrillation or aggregation inhibitors. In a previous study of ours, tau was used as a protein model for fibrillation^22^, allowing a direct comparison between the previously obtained tau data and the current α-Syn data in the presence of GNP-BA. While tau and α-Syn are associated with different neurodegenerative diseases, their aggregation mechanisms share similarities.

Analyzing the effect of GNP-BA on both α-Syn and tau, the results from ThT, CD, and AFM collectively provided evidence that GNP-BA has the potential to prevent fibril formation. In the ThT assay, α-Syn exhibited a slightly prolonged lag time in the presence of GNP-BA, indicating delayed fibrillation. AFM analysis revealed that α-Syn fibrils were fewer in number and displayed branched morphological features, whereas tau fibrils appeared shorter and had a straight morphology in the presence of GNP-BA. Far UV-CD spectrum further supported these findings, showing that when comparing both proteins to their respective fibril controls, α-Syn showed a lower β-sheet content than the tau protein in the presence of GNP-BA. Perhaps we can conclude that GNP-BA is more effective on α-Syn. A deeper understanding of these mechanisms could contribute to identifying novel therapeutic targets for various neurodegenerative diseases.

## Conclusion

PD is characterized by the accumulation of toxic oligomeric and fibrillar species formed by monomeric α-Syn. Certain NPs have been shown to promote protein aggregation, while others have been found to prevent this process^62^. In the current study, we investigated the effects of these opposing behaviors using GNPs conjugated with BA, synthesized through two different methods: citrate-capped GNPs (C-G) covalently conjugated to BA (C-G-BA), and Triton X-100 capped GNPs (U-G) with non-covalent interaction with BA (U-G-BA). Among these, the photochemically synthesised, non-covalent GNP-BA conjugates showed the most significant inhibitory effects on α-Syn aggregation kinetics.

These findings demonstrate that the synthesis method and conjugation type critically influence the therapeutic potential of GNP-based nanoplatforms. By enhancing the solubility, stability, and functional delivery of BA, our approach addresses key pharmacokinetic limitations associated with natural compounds. Furthermore, given emerging evidence that BA-capped GNPs may modulate microbial amyloids and gut microbiota, this study extends its translational value to potential gut–brain axis interventions.

While these in vitro findings are promising, future in vivo studies and molecular investigations will be necessary to confirm the therapeutic efficacy and safety of GNP-BA conjugates and to clarify their mechanism of action against α-Syn aggregation. Overall, our results provide a foundation for the further development of functionalized GNPs as versatile and biocompatible tools for targeting neurodegenerative diseases, with potential applications extending beyond PD.

## Electronic supplementary material

Below is the link to the electronic supplementary material.

Supplementary Material 1

Supplementary Material 2

Supplementary Material 3

Supplementary Material 4

Supplementary Material 5

## Data Availability

The datasets used and/or analyzed during the current study available from the corresponding author on reasonable request.
