# Supplementary figures and images for: Photochemically synthesized gold nanoparticles conjugated with Boswellic acid inhibit alpha synuclein aggregation and delay fibrillation kinetics
Source: Sci Rep. 2025 Jul 17;15:25886. doi: 10.1038/s41598-025-11107-6 (PMC12267561; doi:10.1038/s41598-025-11107-6)

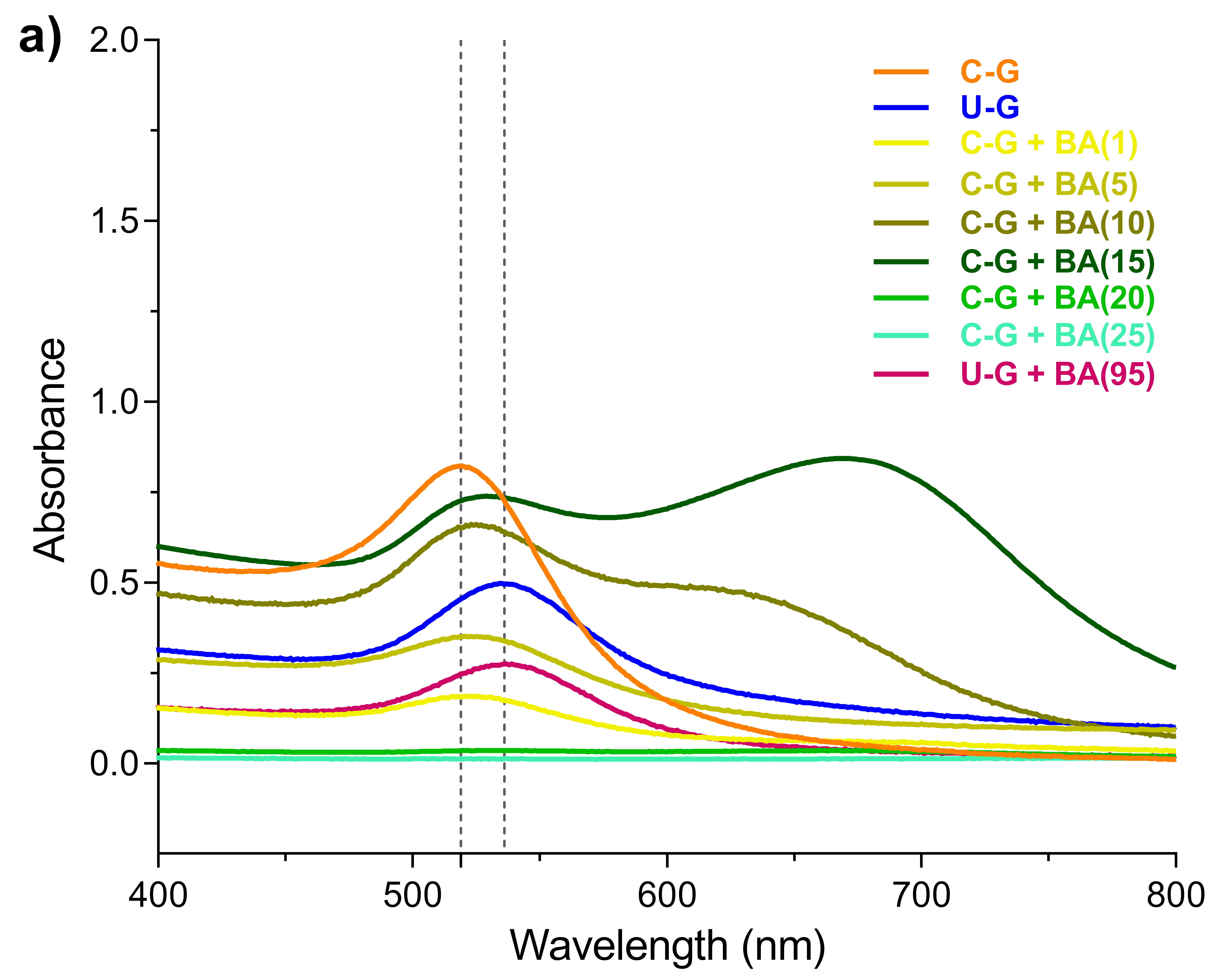

Supplement: Supplementary file 2 — Supplementary Material 2 [file 41598_2025_11107_MOESM2_ESM.png]
