# Supplementary material for: Photochemically synthesized gold nanoparticles conjugated with Boswellic acid inhibit alpha synuclein aggregation and delay fibrillation kinetics
Source: Sci Rep. 2025 Jul 17;15:25886. doi: 10.1038/s41598-025-11107-6 (PMC12267561; doi:10.1038/s41598-025-11107-6)
Supplement: Supplementary file 3 — Supplementary Material 3 [file 41598_2025_11107_MOESM3_ESM.docx]

**SUPPORTING INFORMATION**

**Photochemically Synthesized Gold Nanoparticles Conjugated with Boswellic Acid Inhibit Alpha Synuclein Aggregation and Delay Fibrillation Kinetics**

Masoumeh Gharb^1^, Farima Mozafari^2^, Payam Arghavani^1^, Ali Akbar Saboury^1^, and Gholamhossein Riazi^1,*^

^1^ Institute of Biochemistry and Biophysics, University of Tehran, Tehran 14176-14335, Iran

^2^ School of Chemical Engineering, University of Birmingham, Birmingham, B15 2TT, UK


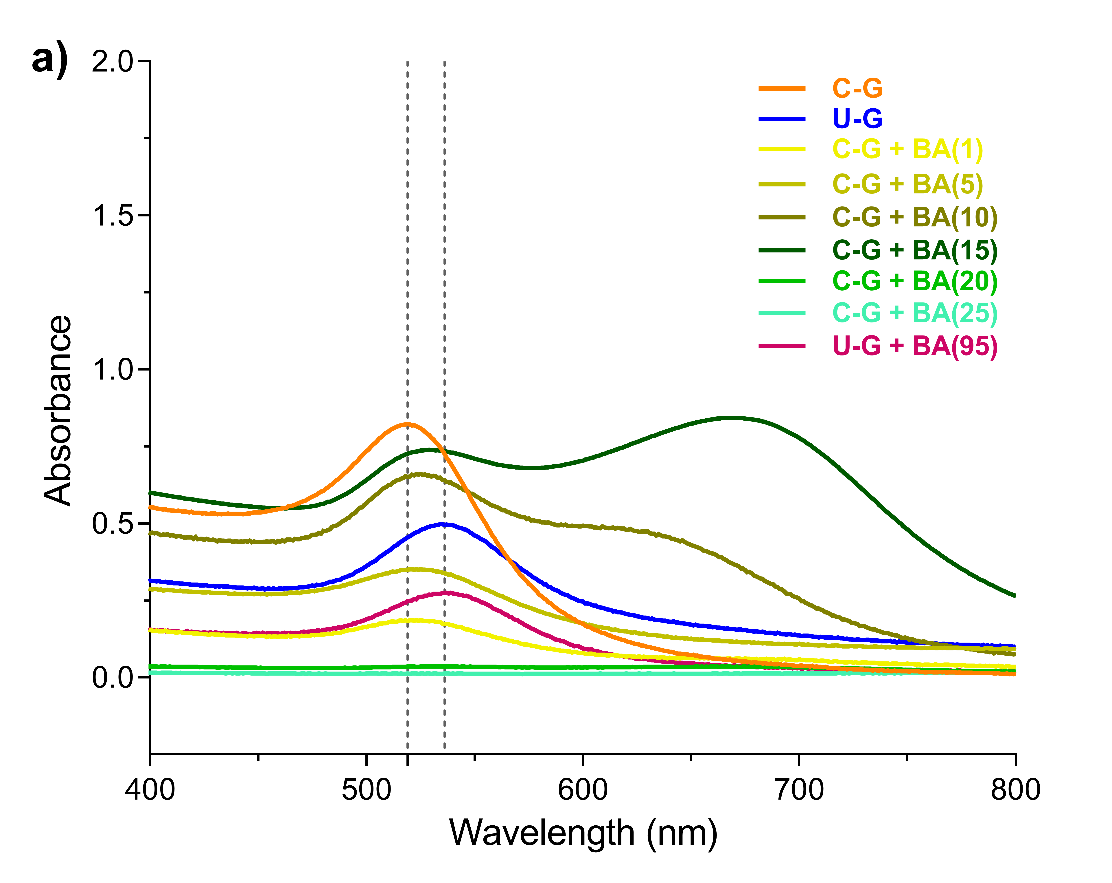


**Supplementary Figure 1. (a) UV−vis absorption spectra distribution.**
