# Supplementary material for: Photochemically synthesized gold nanoparticles conjugated with Boswellic acid inhibit alpha synuclein aggregation and delay fibrillation kinetics
Source: Sci Rep. 2025 Jul 17;15:25886. doi: 10.1038/s41598-025-11107-6 (PMC12267561; doi:10.1038/s41598-025-11107-6)
Supplement: Supplementary file 5 — Supplementary Material 5 [file 41598_2025_11107_MOESM5_ESM.docx]

| λ(nm) | C-G | C-G + BA(1) | C-G + BA(5) | C-G + BA(10) | C-G + BA(15) | C-G + BA(20) | C-G + BA(25) | U-G + BA(95) | U-G |
| --- | --- | --- | --- | --- | --- | --- | --- | --- | --- |
| 800 | 0.01131 | 0.03436 | 0.09236 | 0.07566 | 0.26462 | 0.01897 | 0.01373 | 0.01854 | 0.09916 |
| 799 | 0.0105 | 0.03357 | 0.09264 | 0.07497 | 0.26691 | 0.01897 | 0.01368 | 0.02089 | 0.10087 |
| 798 | 0.01139 | 0.03435 | 0.09129 | 0.07565 | 0.27003 | 0.01884 | 0.0133 | 0.01856 | 0.09865 |
| 797 | 0.01057 | 0.03483 | 0.0922 | 0.07765 | 0.27268 | 0.01902 | 0.01308 | 0.02081 | 0.1024 |
| 796 | 0.0129 | 0.03661 | 0.09349 | 0.07612 | 0.27601 | 0.01905 | 0.01368 | 0.02015 | 0.10091 |
| 795 | 0.01097 | 0.03445 | 0.09306 | 0.07766 | 0.27921 | 0.01923 | 0.01337 | 0.01767 | 0.10124 |
| 794 | 0.01163 | 0.03543 | 0.09372 | 0.07904 | 0.28201 | 0.01914 | 0.01288 | 0.01944 | 0.10176 |
| 793 | 0.01178 | 0.03487 | 0.09252 | 0.08047 | 0.28535 | 0.01904 | 0.01381 | 0.01906 | 0.10131 |
| 792 | 0.0143 | 0.0373 | 0.09303 | 0.07999 | 0.28898 | 0.02002 | 0.0137 | 0.01955 | 0.10262 |
| 791 | 0.01305 | 0.03484 | 0.09237 | 0.08244 | 0.29231 | 0.01921 | 0.01343 | 0.02004 | 0.10235 |
| 790 | 0.01306 | 0.03645 | 0.09213 | 0.07789 | 0.29559 | 0.01985 | 0.01374 | 0.02056 | 0.10179 |
| 789 | 0.01414 | 0.0369 | 0.09367 | 0.08428 | 0.29911 | 0.01966 | 0.01356 | 0.01992 | 0.10131 |
| 788 | 0.01421 | 0.03769 | 0.0938 | 0.08214 | 0.30215 | 0.02001 | 0.01372 | 0.01862 | 0.10466 |
| 787 | 0.01381 | 0.03698 | 0.09449 | 0.0845 | 0.30619 | 0.01984 | 0.01382 | 0.01801 | 0.10119 |
| 786 | 0.01364 | 0.03725 | 0.09471 | 0.08361 | 0.31002 | 0.02025 | 0.01396 | 0.0188 | 0.10381 |
| 785 | 0.01324 | 0.03642 | 0.09276 | 0.0859 | 0.31363 | 0.01996 | 0.01375 | 0.02007 | 0.10249 |
| 784 | 0.01324 | 0.03765 | 0.09276 | 0.08696 | 0.31688 | 0.02011 | 0.01423 | 0.02 | 0.10553 |
| 783 | 0.01411 | 0.03743 | 0.09396 | 0.08573 | 0.3204 | 0.02012 | 0.01389 | 0.01964 | 0.10221 |
| 782 | 0.01352 | 0.03653 | 0.09402 | 0.09006 | 0.32442 | 0.01992 | 0.01398 | 0.01741 | 0.10401 |
| 781 | 0.01407 | 0.03762 | 0.09378 | 0.0896 | 0.32839 | 0.02071 | 0.01364 | 0.02115 | 0.10401 |
| 780 | 0.01448 | 0.03706 | 0.0936 | 0.09179 | 0.33268 | 0.02066 | 0.01418 | 0.01973 | 0.10394 |
| 779 | 0.01513 | 0.03779 | 0.09476 | 0.09171 | 0.33581 | 0.02062 | 0.01393 | 0.02029 | 0.10437 |
| 778 | 0.01519 | 0.03775 | 0.09417 | 0.09329 | 0.34057 | 0.02078 | 0.01399 | 0.01988 | 0.10602 |
| 777 | 0.01472 | 0.03819 | 0.09472 | 0.09304 | 0.34465 | 0.0213 | 0.01398 | 0.02073 | 0.10557 |
| 776 | 0.01443 | 0.03987 | 0.09431 | 0.09519 | 0.34881 | 0.02147 | 0.01408 | 0.02044 | 0.10592 |
| 775 | 0.01468 | 0.03848 | 0.09443 | 0.09558 | 0.35253 | 0.02112 | 0.01425 | 0.02107 | 0.10521 |
| 774 | 0.01586 | 0.03966 | 0.09428 | 0.0956 | 0.35732 | 0.02115 | 0.01365 | 0.02071 | 0.10611 |
| 773 | 0.01531 | 0.03924 | 0.09448 | 0.09664 | 0.36155 | 0.02195 | 0.0143 | 0.02285 | 0.1057 |
| 772 | 0.01566 | 0.0393 | 0.0961 | 0.09717 | 0.36592 | 0.02123 | 0.01411 | 0.01983 | 0.10631 |
| 771 | 0.01613 | 0.03954 | 0.09526 | 0.09824 | 0.3704 | 0.02148 | 0.01376 | 0.0208 | 0.10508 |
| 770 | 0.01645 | 0.04033 | 0.09451 | 0.10021 | 0.37548 | 0.02149 | 0.01481 | 0.02104 | 0.10615 |
| 769 | 0.01586 | 0.04087 | 0.09526 | 0.10084 | 0.37996 | 0.02229 | 0.0141 | 0.02232 | 0.10802 |
| 768 | 0.01555 | 0.04111 | 0.0954 | 0.10469 | 0.38478 | 0.02173 | 0.01456 | 0.02123 | 0.10752 |
| 767 | 0.01754 | 0.0412 | 0.0963 | 0.10448 | 0.38924 | 0.02232 | 0.01471 | 0.02102 | 0.10827 |
| 766 | 0.01608 | 0.04025 | 0.09495 | 0.10532 | 0.39387 | 0.02193 | 0.01409 | 0.02313 | 0.10902 |
| 765 | 0.01711 | 0.03988 | 0.0956 | 0.10164 | 0.39911 | 0.02267 | 0.01405 | 0.02115 | 0.10957 |
| 764 | 0.01651 | 0.03979 | 0.09524 | 0.1072 | 0.40425 | 0.0224 | 0.01488 | 0.02209 | 0.10709 |
| 763 | 0.01617 | 0.04292 | 0.09468 | 0.10864 | 0.40886 | 0.02233 | 0.01407 | 0.02129 | 0.10899 |
| 762 | 0.01668 | 0.03976 | 0.09627 | 0.10935 | 0.41448 | 0.0232 | 0.01466 | 0.02085 | 0.11047 |
| 761 | 0.01785 | 0.03991 | 0.09574 | 0.11104 | 0.4187 | 0.02259 | 0.01442 | 0.02338 | 0.10887 |
| 760 | 0.01714 | 0.04072 | 0.09655 | 0.11127 | 0.42445 | 0.02355 | 0.01418 | 0.02338 | 0.11026 |
| 759 | 0.0165 | 0.04102 | 0.09488 | 0.11256 | 0.42958 | 0.02293 | 0.01391 | 0.02117 | 0.11114 |
| 758 | 0.01649 | 0.04126 | 0.09551 | 0.11595 | 0.43486 | 0.02346 | 0.01404 | 0.02291 | 0.11107 |
| 757 | 0.01795 | 0.0402 | 0.09512 | 0.11659 | 0.44072 | 0.02364 | 0.01375 | 0.02227 | 0.11129 |
| 756 | 0.01787 | 0.04125 | 0.09518 | 0.11562 | 0.44564 | 0.02404 | 0.01332 | 0.02409 | 0.11186 |
| 755 | 0.01957 | 0.04258 | 0.09645 | 0.11899 | 0.45125 | 0.02409 | 0.01343 | 0.02247 | 0.11214 |
| 754 | 0.01949 | 0.0417 | 0.09598 | 0.11874 | 0.45708 | 0.02448 | 0.01389 | 0.02222 | 0.11304 |
| 753 | 0.02005 | 0.04278 | 0.09672 | 0.12053 | 0.4622 | 0.02385 | 0.01347 | 0.02513 | 0.11293 |
| 752 | 0.02048 | 0.04334 | 0.09669 | 0.12432 | 0.4678 | 0.02481 | 0.01352 | 0.02374 | 0.11245 |
| 751 | 0.01897 | 0.04316 | 0.09673 | 0.12203 | 0.47438 | 0.02459 | 0.01345 | 0.02178 | 0.11264 |
| 750 | 0.0193 | 0.04423 | 0.09696 | 0.12711 | 0.48018 | 0.02461 | 0.01341 | 0.02318 | 0.11258 |
| 749 | 0.01982 | 0.04412 | 0.09739 | 0.12964 | 0.486 | 0.02472 | 0.01346 | 0.02215 | 0.1125 |
| 748 | 0.0198 | 0.04351 | 0.09753 | 0.1287 | 0.49181 | 0.02471 | 0.01324 | 0.02408 | 0.11357 |
| 747 | 0.02017 | 0.04411 | 0.09664 | 0.12976 | 0.49816 | 0.02555 | 0.01314 | 0.02343 | 0.1148 |
| 746 | 0.0206 | 0.04401 | 0.09743 | 0.13256 | 0.50374 | 0.02528 | 0.01363 | 0.02409 | 0.11663 |
| 745 | 0.01999 | 0.04425 | 0.0974 | 0.13601 | 0.51003 | 0.02592 | 0.01363 | 0.02477 | 0.11639 |
| 744 | 0.02215 | 0.04456 | 0.09842 | 0.1363 | 0.51631 | 0.02572 | 0.01321 | 0.02217 | 0.11326 |
| 743 | 0.02001 | 0.04456 | 0.09847 | 0.13904 | 0.52228 | 0.02595 | 0.01344 | 0.02501 | 0.11719 |
| 742 | 0.01952 | 0.04375 | 0.09748 | 0.14032 | 0.5289 | 0.02592 | 0.01346 | 0.02311 | 0.11537 |
| 741 | 0.02243 | 0.04476 | 0.0984 | 0.14335 | 0.53481 | 0.0258 | 0.01336 | 0.02532 | 0.11815 |
| 740 | 0.0207 | 0.04448 | 0.09724 | 0.14385 | 0.54079 | 0.02652 | 0.01288 | 0.02482 | 0.11744 |
| 739 | 0.02164 | 0.04478 | 0.09864 | 0.14453 | 0.54684 | 0.02602 | 0.01295 | 0.02399 | 0.11702 |
| 738 | 0.024 | 0.04464 | 0.09902 | 0.14725 | 0.55361 | 0.0267 | 0.01345 | 0.02384 | 0.11749 |
| 737 | 0.02251 | 0.0449 | 0.09861 | 0.15101 | 0.5598 | 0.02653 | 0.01284 | 0.02677 | 0.11772 |
| 736 | 0.02183 | 0.04635 | 0.09928 | 0.151 | 0.56625 | 0.0274 | 0.01371 | 0.02479 | 0.11898 |
| 735 | 0.02412 | 0.04672 | 0.10048 | 0.15577 | 0.57332 | 0.02729 | 0.01329 | 0.02417 | 0.11999 |
| 734 | 0.0226 | 0.04729 | 0.1 | 0.15588 | 0.57906 | 0.02724 | 0.01298 | 0.02482 | 0.12019 |
| 733 | 0.02353 | 0.04759 | 0.09957 | 0.15621 | 0.58546 | 0.02797 | 0.01346 | 0.02606 | 0.11926 |
| 732 | 0.02444 | 0.04758 | 0.09879 | 0.16063 | 0.59202 | 0.02773 | 0.01331 | 0.02509 | 0.11975 |
| 731 | 0.02485 | 0.04784 | 0.10011 | 0.16254 | 0.5982 | 0.02814 | 0.01357 | 0.02517 | 0.12199 |
| 730 | 0.02457 | 0.0481 | 0.10015 | 0.16684 | 0.60548 | 0.0279 | 0.01285 | 0.02434 | 0.12163 |
| 729 | 0.02539 | 0.04841 | 0.10058 | 0.1674 | 0.61163 | 0.02825 | 0.01321 | 0.02753 | 0.12145 |
| 728 | 0.02566 | 0.04767 | 0.10146 | 0.16611 | 0.61895 | 0.02862 | 0.01352 | 0.02698 | 0.12159 |
| 727 | 0.02542 | 0.04672 | 0.10065 | 0.17334 | 0.62461 | 0.02923 | 0.01349 | 0.02649 | 0.12206 |
| 726 | 0.02566 | 0.04925 | 0.10056 | 0.17469 | 0.63146 | 0.02894 | 0.01372 | 0.02473 | 0.12351 |
| 725 | 0.02573 | 0.04696 | 0.101 | 0.1771 | 0.63766 | 0.02892 | 0.01339 | 0.02744 | 0.1221 |
| 724 | 0.02656 | 0.04932 | 0.10228 | 0.17722 | 0.64415 | 0.02983 | 0.01361 | 0.02662 | 0.12252 |
| 723 | 0.02682 | 0.04949 | 0.10097 | 0.18292 | 0.65032 | 0.02929 | 0.01309 | 0.02555 | 0.12346 |
| 722 | 0.0254 | 0.04884 | 0.10064 | 0.18547 | 0.65671 | 0.02982 | 0.01339 | 0.02516 | 0.12348 |
| 721 | 0.02839 | 0.04885 | 0.10099 | 0.18782 | 0.66228 | 0.02939 | 0.01295 | 0.02724 | 0.12613 |
| 720 | 0.02714 | 0.04907 | 0.10052 | 0.18786 | 0.66883 | 0.03007 | 0.013 | 0.02762 | 0.1248 |
| 719 | 0.02784 | 0.04957 | 0.10191 | 0.19386 | 0.67491 | 0.03008 | 0.01343 | 0.0261 | 0.12649 |
| 718 | 0.02943 | 0.0511 | 0.10289 | 0.19636 | 0.68327 | 0.03085 | 0.01327 | 0.02627 | 0.12739 |
| 717 | 0.03045 | 0.05292 | 0.10401 | 0.19716 | 0.68747 | 0.0304 | 0.01321 | 0.02761 | 0.12884 |
| 716 | 0.0295 | 0.05175 | 0.10368 | 0.20091 | 0.69349 | 0.03071 | 0.01305 | 0.02599 | 0.12796 |
| 715 | 0.0301 | 0.05008 | 0.10304 | 0.20124 | 0.69948 | 0.03092 | 0.01291 | 0.02713 | 0.12784 |
| 714 | 0.03034 | 0.05113 | 0.10221 | 0.20699 | 0.70492 | 0.03097 | 0.01279 | 0.02847 | 0.12927 |
| 713 | 0.0315 | 0.05139 | 0.10252 | 0.21041 | 0.71134 | 0.03121 | 0.01299 | 0.02779 | 0.12882 |
| 712 | 0.03421 | 0.05349 | 0.10428 | 0.21148 | 0.71647 | 0.03137 | 0.01265 | 0.02862 | 0.12906 |
| 711 | 0.03256 | 0.05387 | 0.10411 | 0.21709 | 0.72232 | 0.03123 | 0.01277 | 0.02866 | 0.12989 |
| 710 | 0.03366 | 0.05385 | 0.10441 | 0.21975 | 0.72786 | 0.0313 | 0.01285 | 0.02799 | 0.13074 |
| 709 | 0.0339 | 0.05352 | 0.10345 | 0.22341 | 0.73318 | 0.03146 | 0.01287 | 0.02857 | 0.13196 |
| 708 | 0.035 | 0.05479 | 0.10451 | 0.22392 | 0.73847 | 0.03157 | 0.01299 | 0.02771 | 0.12925 |
| 707 | 0.0346 | 0.0549 | 0.10388 | 0.22831 | 0.74366 | 0.032 | 0.01267 | 0.02733 | 0.13165 |
| 706 | 0.03799 | 0.05685 | 0.10596 | 0.23338 | 0.74919 | 0.03221 | 0.0128 | 0.02962 | 0.1322 |
| 705 | 0.03572 | 0.05558 | 0.1052 | 0.23363 | 0.75421 | 0.03238 | 0.01297 | 0.02922 | 0.1326 |
| 704 | 0.03617 | 0.05561 | 0.10675 | 0.23907 | 0.7587 | 0.03275 | 0.01268 | 0.02907 | 0.13373 |
| 703 | 0.03596 | 0.05582 | 0.106 | 0.24145 | 0.76331 | 0.03216 | 0.01266 | 0.02804 | 0.13482 |
| 702 | 0.0364 | 0.05726 | 0.10686 | 0.24711 | 0.76865 | 0.03293 | 0.01276 | 0.03102 | 0.13573 |
| 701 | 0.03834 | 0.05682 | 0.10683 | 0.24774 | 0.77295 | 0.03341 | 0.01266 | 0.02904 | 0.13531 |
| 700 | 0.03747 | 0.05895 | 0.10948 | 0.25622 | 0.77704 | 0.03315 | 0.01281 | 0.02711 | 0.13451 |
| 699 | 0.03693 | 0.05591 | 0.10703 | 0.25962 | 0.78171 | 0.03301 | 0.01289 | 0.03196 | 0.13636 |
| 698 | 0.03791 | 0.05548 | 0.10662 | 0.26194 | 0.78555 | 0.0329 | 0.01257 | 0.0297 | 0.13706 |
| 697 | 0.03847 | 0.05656 | 0.1075 | 0.26409 | 0.7894 | 0.03278 | 0.01282 | 0.03071 | 0.13918 |
| 696 | 0.0399 | 0.05752 | 0.10795 | 0.26958 | 0.79323 | 0.03338 | 0.01254 | 0.02952 | 0.13715 |
| 695 | 0.03953 | 0.058 | 0.10785 | 0.27119 | 0.79655 | 0.03333 | 0.01232 | 0.03134 | 0.1402 |
| 694 | 0.04075 | 0.05675 | 0.10821 | 0.27284 | 0.80041 | 0.03328 | 0.01256 | 0.03164 | 0.14013 |
| 693 | 0.04113 | 0.06029 | 0.10902 | 0.27978 | 0.80379 | 0.03368 | 0.01225 | 0.03242 | 0.13927 |
| 692 | 0.04038 | 0.05813 | 0.1077 | 0.28576 | 0.80739 | 0.03279 | 0.01183 | 0.03225 | 0.14063 |
| 691 | 0.04342 | 0.05893 | 0.10879 | 0.28694 | 0.81078 | 0.03389 | 0.01266 | 0.03153 | 0.13967 |
| 690 | 0.04235 | 0.05736 | 0.1093 | 0.29205 | 0.81399 | 0.03421 | 0.0122 | 0.03383 | 0.1425 |
| 689 | 0.04194 | 0.05638 | 0.10777 | 0.29497 | 0.81688 | 0.03413 | 0.01263 | 0.03048 | 0.14436 |
| 688 | 0.04299 | 0.05872 | 0.10967 | 0.30159 | 0.82042 | 0.03422 | 0.01244 | 0.03256 | 0.14097 |
| 687 | 0.04447 | 0.05799 | 0.11076 | 0.30322 | 0.82232 | 0.03479 | 0.01291 | 0.03117 | 0.14413 |
| 686 | 0.04331 | 0.05778 | 0.11001 | 0.30662 | 0.82515 | 0.03464 | 0.01241 | 0.03295 | 0.14458 |
| 685 | 0.0444 | 0.05971 | 0.11044 | 0.31034 | 0.82706 | 0.03466 | 0.01298 | 0.03114 | 0.14475 |
| 684 | 0.04615 | 0.05754 | 0.11174 | 0.3126 | 0.82946 | 0.03494 | 0.01282 | 0.03271 | 0.14397 |
| 683 | 0.04522 | 0.05757 | 0.11052 | 0.32025 | 0.83099 | 0.03468 | 0.01279 | 0.03167 | 0.14601 |
| 682 | 0.04669 | 0.05911 | 0.11157 | 0.32139 | 0.83307 | 0.03448 | 0.01267 | 0.03376 | 0.14783 |
| 681 | 0.0474 | 0.05656 | 0.11293 | 0.3247 | 0.83449 | 0.03481 | 0.01217 | 0.03362 | 0.14701 |
| 680 | 0.04874 | 0.06015 | 0.11191 | 0.32815 | 0.8358 | 0.03481 | 0.01213 | 0.03273 | 0.14862 |
| 679 | 0.04943 | 0.05863 | 0.11371 | 0.33099 | 0.83715 | 0.03474 | 0.01248 | 0.03294 | 0.14933 |
| 678 | 0.04723 | 0.0582 | 0.11125 | 0.33724 | 0.83794 | 0.035 | 0.01246 | 0.03573 | 0.1489 |
| 677 | 0.04902 | 0.05976 | 0.11509 | 0.34226 | 0.83896 | 0.03509 | 0.01273 | 0.03535 | 0.15126 |
| 676 | 0.05051 | 0.06023 | 0.11408 | 0.34147 | 0.83946 | 0.03459 | 0.01215 | 0.03493 | 0.15116 |
| 675 | 0.05237 | 0.06108 | 0.11334 | 0.34845 | 0.84049 | 0.03493 | 0.01232 | 0.03564 | 0.15175 |
| 674 | 0.05086 | 0.05964 | 0.11407 | 0.35282 | 0.8413 | 0.03508 | 0.01225 | 0.03678 | 0.15445 |
| 673 | 0.05235 | 0.05983 | 0.1138 | 0.35281 | 0.84196 | 0.03445 | 0.01157 | 0.03694 | 0.15285 |
| 672 | 0.05296 | 0.06172 | 0.11494 | 0.36048 | 0.8428 | 0.03449 | 0.01238 | 0.03524 | 0.15513 |
| 671 | 0.05404 | 0.061 | 0.11641 | 0.36432 | 0.84276 | 0.03479 | 0.01213 | 0.03607 | 0.15507 |
| 670 | 0.05483 | 0.06048 | 0.11557 | 0.36648 | 0.8433 | 0.03448 | 0.01221 | 0.03674 | 0.15491 |
| 669 | 0.05429 | 0.059 | 0.11575 | 0.37051 | 0.84257 | 0.03423 | 0.01182 | 0.03979 | 0.1568 |
| 668 | 0.05731 | 0.06142 | 0.11651 | 0.37274 | 0.84297 | 0.03553 | 0.012 | 0.03695 | 0.15692 |
| 667 | 0.05667 | 0.06132 | 0.11706 | 0.37769 | 0.84264 | 0.0347 | 0.01209 | 0.03598 | 0.15657 |
| 666 | 0.05744 | 0.06025 | 0.11752 | 0.3814 | 0.84264 | 0.03529 | 0.01249 | 0.03773 | 0.15869 |
| 665 | 0.05739 | 0.06011 | 0.11716 | 0.38494 | 0.8422 | 0.03478 | 0.01247 | 0.03916 | 0.15818 |
| 664 | 0.05956 | 0.06086 | 0.1177 | 0.39015 | 0.84131 | 0.03543 | 0.01179 | 0.03816 | 0.15888 |
| 663 | 0.05883 | 0.06149 | 0.12024 | 0.39065 | 0.84 | 0.03459 | 0.01174 | 0.03708 | 0.16045 |
| 662 | 0.06048 | 0.06226 | 0.11929 | 0.39497 | 0.84017 | 0.03469 | 0.01213 | 0.03978 | 0.16162 |
| 661 | 0.06006 | 0.06152 | 0.11947 | 0.40001 | 0.83891 | 0.0353 | 0.0124 | 0.04002 | 0.16167 |
| 660 | 0.06182 | 0.06052 | 0.12007 | 0.40182 | 0.83755 | 0.03504 | 0.01222 | 0.0398 | 0.16357 |
| 659 | 0.06331 | 0.06227 | 0.12084 | 0.40348 | 0.83691 | 0.03512 | 0.01185 | 0.03842 | 0.16326 |
| 658 | 0.06525 | 0.06159 | 0.1214 | 0.40693 | 0.83589 | 0.03448 | 0.01152 | 0.04024 | 0.16507 |
| 657 | 0.06558 | 0.0624 | 0.12132 | 0.41095 | 0.83432 | 0.035 | 0.01165 | 0.04228 | 0.16566 |
| 656 | 0.06637 | 0.06202 | 0.12202 | 0.411 | 0.83269 | 0.03443 | 0.01161 | 0.04064 | 0.16612 |
| 655 | 0.06722 | 0.06357 | 0.12291 | 0.41696 | 0.83175 | 0.03477 | 0.01187 | 0.04143 | 0.16723 |
| 654 | 0.06799 | 0.06101 | 0.12199 | 0.41963 | 0.8302 | 0.03466 | 0.01132 | 0.04256 | 0.16733 |
| 653 | 0.07052 | 0.06346 | 0.12351 | 0.42182 | 0.82813 | 0.03486 | 0.01131 | 0.04374 | 0.1672 |
| 652 | 0.07037 | 0.06286 | 0.12301 | 0.42463 | 0.82691 | 0.03483 | 0.01164 | 0.04354 | 0.16879 |
| 651 | 0.0729 | 0.06294 | 0.12377 | 0.42748 | 0.82536 | 0.03456 | 0.01172 | 0.04371 | 0.17082 |
| 650 | 0.07188 | 0.06225 | 0.12566 | 0.42994 | 0.82323 | 0.03461 | 0.01182 | 0.04562 | 0.16915 |
| 649 | 0.07311 | 0.06299 | 0.12512 | 0.43348 | 0.82212 | 0.03452 | 0.01161 | 0.04553 | 0.17109 |
| 648 | 0.07477 | 0.06341 | 0.12724 | 0.43542 | 0.8195 | 0.03451 | 0.01156 | 0.0461 | 0.17245 |
| 647 | 0.07563 | 0.06358 | 0.12544 | 0.43993 | 0.81766 | 0.03428 | 0.01155 | 0.04536 | 0.17337 |
| 646 | 0.0763 | 0.06383 | 0.1285 | 0.44482 | 0.81528 | 0.03413 | 0.01157 | 0.04644 | 0.1742 |
| 645 | 0.07744 | 0.06197 | 0.12697 | 0.44132 | 0.81348 | 0.03418 | 0.01186 | 0.04763 | 0.17543 |
| 644 | 0.07733 | 0.06341 | 0.12844 | 0.44522 | 0.81121 | 0.03429 | 0.0119 | 0.04761 | 0.17615 |
| 643 | 0.07847 | 0.06174 | 0.12887 | 0.4464 | 0.8091 | 0.0343 | 0.01192 | 0.04748 | 0.17794 |
| 642 | 0.08081 | 0.06225 | 0.12828 | 0.44937 | 0.80723 | 0.03419 | 0.01208 | 0.0468 | 0.17785 |
| 641 | 0.08269 | 0.06295 | 0.12935 | 0.45023 | 0.80456 | 0.03439 | 0.01155 | 0.05068 | 0.18183 |
| 640 | 0.08338 | 0.06367 | 0.13083 | 0.45428 | 0.80193 | 0.03395 | 0.01184 | 0.04948 | 0.18109 |
| 639 | 0.08509 | 0.06152 | 0.13268 | 0.45519 | 0.79976 | 0.03412 | 0.01169 | 0.04929 | 0.18032 |
| 638 | 0.08589 | 0.06352 | 0.1319 | 0.4579 | 0.79742 | 0.03398 | 0.01151 | 0.05097 | 0.1813 |
| 637 | 0.08802 | 0.06483 | 0.13284 | 0.45799 | 0.79483 | 0.03433 | 0.01169 | 0.04981 | 0.18391 |
| 636 | 0.0892 | 0.06461 | 0.13523 | 0.46113 | 0.79259 | 0.03396 | 0.0116 | 0.0513 | 0.18472 |
| 635 | 0.09132 | 0.06383 | 0.1351 | 0.46485 | 0.79041 | 0.03365 | 0.01145 | 0.05257 | 0.1859 |
| 634 | 0.09299 | 0.06465 | 0.13602 | 0.46102 | 0.78737 | 0.03367 | 0.01157 | 0.05367 | 0.18684 |
| 633 | 0.09467 | 0.06591 | 0.13782 | 0.46687 | 0.78489 | 0.03369 | 0.0116 | 0.05303 | 0.18643 |
| 632 | 0.09453 | 0.06652 | 0.13745 | 0.46695 | 0.78277 | 0.03359 | 0.01145 | 0.05592 | 0.18783 |
| 631 | 0.09795 | 0.06584 | 0.13808 | 0.46726 | 0.78031 | 0.03352 | 0.01217 | 0.05588 | 0.19093 |
| 630 | 0.09887 | 0.06474 | 0.13801 | 0.47319 | 0.77768 | 0.03373 | 0.01208 | 0.05765 | 0.18919 |
| 629 | 0.10093 | 0.06632 | 0.14097 | 0.47316 | 0.77481 | 0.03368 | 0.01191 | 0.0575 | 0.19244 |
| 628 | 0.1027 | 0.067 | 0.14267 | 0.47314 | 0.77229 | 0.03354 | 0.01178 | 0.05983 | 0.19331 |
| 627 | 0.10359 | 0.0655 | 0.14171 | 0.47417 | 0.76997 | 0.03345 | 0.01179 | 0.05962 | 0.19382 |
| 626 | 0.10637 | 0.06804 | 0.14249 | 0.47275 | 0.76707 | 0.03366 | 0.01242 | 0.06133 | 0.19489 |
| 625 | 0.10827 | 0.0667 | 0.14385 | 0.47534 | 0.76421 | 0.03312 | 0.01188 | 0.05917 | 0.19754 |
| 624 | 0.10963 | 0.0672 | 0.14739 | 0.47489 | 0.76187 | 0.0329 | 0.01185 | 0.06084 | 0.19829 |
| 623 | 0.11137 | 0.0673 | 0.14505 | 0.47787 | 0.75912 | 0.03352 | 0.01202 | 0.06127 | 0.2009 |
| 622 | 0.11432 | 0.06905 | 0.14622 | 0.48164 | 0.7569 | 0.03309 | 0.01166 | 0.06538 | 0.20238 |
| 621 | 0.11529 | 0.06819 | 0.14745 | 0.47944 | 0.75342 | 0.03302 | 0.01157 | 0.06546 | 0.20224 |
| 620 | 0.11689 | 0.06695 | 0.14947 | 0.47808 | 0.7507 | 0.03299 | 0.01216 | 0.06498 | 0.20462 |
| 619 | 0.12063 | 0.06917 | 0.15002 | 0.48287 | 0.74894 | 0.03298 | 0.01129 | 0.06598 | 0.20602 |
| 618 | 0.12236 | 0.06928 | 0.15026 | 0.48259 | 0.74573 | 0.03265 | 0.01161 | 0.0679 | 0.2078 |
| 617 | 0.12422 | 0.06959 | 0.15113 | 0.48423 | 0.74392 | 0.03313 | 0.01195 | 0.06976 | 0.21052 |
| 616 | 0.12645 | 0.07103 | 0.15343 | 0.48551 | 0.74073 | 0.03258 | 0.01174 | 0.06891 | 0.21129 |
| 615 | 0.12894 | 0.07134 | 0.15531 | 0.48348 | 0.73884 | 0.03251 | 0.0119 | 0.07031 | 0.21158 |
| 614 | 0.13106 | 0.07172 | 0.15389 | 0.48621 | 0.73565 | 0.03262 | 0.01157 | 0.07182 | 0.21516 |
| 613 | 0.13544 | 0.07196 | 0.15601 | 0.48522 | 0.73362 | 0.03233 | 0.01163 | 0.07355 | 0.21428 |
| 612 | 0.13633 | 0.07126 | 0.15898 | 0.48433 | 0.73155 | 0.03287 | 0.01166 | 0.07533 | 0.21849 |
| 611 | 0.13981 | 0.07147 | 0.1607 | 0.48596 | 0.72858 | 0.03251 | 0.01174 | 0.0748 | 0.21988 |
| 610 | 0.14337 | 0.07371 | 0.16068 | 0.48696 | 0.72624 | 0.03278 | 0.01169 | 0.07766 | 0.22074 |
| 609 | 0.14425 | 0.07301 | 0.1619 | 0.49011 | 0.72456 | 0.03224 | 0.01194 | 0.07949 | 0.22402 |
| 608 | 0.14772 | 0.07371 | 0.16362 | 0.4844 | 0.72159 | 0.03264 | 0.01206 | 0.0788 | 0.22561 |
| 607 | 0.15078 | 0.07314 | 0.16389 | 0.48587 | 0.71922 | 0.03207 | 0.0119 | 0.08134 | 0.22704 |
| 606 | 0.1529 | 0.07394 | 0.16545 | 0.48663 | 0.71728 | 0.0323 | 0.01182 | 0.08425 | 0.22865 |
| 605 | 0.15538 | 0.07521 | 0.16765 | 0.48707 | 0.71466 | 0.03239 | 0.01172 | 0.08554 | 0.23276 |
| 604 | 0.16053 | 0.07709 | 0.1689 | 0.49012 | 0.71269 | 0.03244 | 0.01172 | 0.08591 | 0.23318 |
| 603 | 0.16349 | 0.0775 | 0.17076 | 0.49058 | 0.71074 | 0.03234 | 0.01183 | 0.09095 | 0.23491 |
| 602 | 0.16509 | 0.07578 | 0.16974 | 0.49143 | 0.70798 | 0.03191 | 0.01156 | 0.09065 | 0.2384 |
| 601 | 0.16925 | 0.07741 | 0.17403 | 0.49186 | 0.70681 | 0.0324 | 0.01174 | 0.09097 | 0.24051 |
| 600 | 0.17323 | 0.07935 | 0.17601 | 0.49263 | 0.70389 | 0.03177 | 0.01155 | 0.09205 | 0.24073 |
| 599 | 0.17668 | 0.07897 | 0.17666 | 0.48998 | 0.70243 | 0.03217 | 0.01149 | 0.09352 | 0.24613 |
| 598 | 0.17974 | 0.07904 | 0.17826 | 0.49045 | 0.70089 | 0.03241 | 0.01166 | 0.09748 | 0.24761 |
| 597 | 0.18346 | 0.08228 | 0.1802 | 0.49197 | 0.69917 | 0.03222 | 0.01164 | 0.1006 | 0.24851 |
| 596 | 0.18904 | 0.08244 | 0.18156 | 0.49138 | 0.69749 | 0.03241 | 0.01203 | 0.10195 | 0.25222 |
| 595 | 0.191 | 0.08043 | 0.18377 | 0.49254 | 0.69607 | 0.03185 | 0.01178 | 0.10048 | 0.25509 |
| 594 | 0.19738 | 0.08443 | 0.18502 | 0.49177 | 0.69399 | 0.0319 | 0.01125 | 0.10523 | 0.25814 |
| 593 | 0.19969 | 0.08437 | 0.18803 | 0.49171 | 0.69305 | 0.0323 | 0.01152 | 0.10694 | 0.26124 |
| 592 | 0.20531 | 0.08558 | 0.19039 | 0.49609 | 0.69109 | 0.0315 | 0.01117 | 0.1107 | 0.26623 |
| 591 | 0.21094 | 0.0868 | 0.19183 | 0.49431 | 0.68974 | 0.03157 | 0.01115 | 0.11357 | 0.26744 |
| 590 | 0.21453 | 0.08661 | 0.19245 | 0.49581 | 0.68835 | 0.03215 | 0.01136 | 0.11705 | 0.2709 |
| 589 | 0.21747 | 0.08778 | 0.19479 | 0.49436 | 0.68738 | 0.03185 | 0.01158 | 0.11763 | 0.27405 |
| 588 | 0.22304 | 0.08859 | 0.19644 | 0.49367 | 0.68661 | 0.032 | 0.01144 | 0.12129 | 0.27852 |
| 587 | 0.22856 | 0.09027 | 0.19876 | 0.49669 | 0.68508 | 0.03173 | 0.01144 | 0.12213 | 0.28164 |
| 586 | 0.23414 | 0.09112 | 0.20136 | 0.49784 | 0.68455 | 0.03185 | 0.01139 | 0.12386 | 0.28469 |
| 585 | 0.24131 | 0.09331 | 0.20299 | 0.50125 | 0.68348 | 0.03155 | 0.01146 | 0.12867 | 0.28804 |
| 584 | 0.24419 | 0.09303 | 0.20525 | 0.5032 | 0.68229 | 0.03133 | 0.01142 | 0.13181 | 0.29295 |
| 583 | 0.25176 | 0.09436 | 0.20673 | 0.50172 | 0.68207 | 0.03185 | 0.01099 | 0.13546 | 0.29354 |
| 582 | 0.2568 | 0.09597 | 0.20944 | 0.50287 | 0.68107 | 0.03174 | 0.01103 | 0.13697 | 0.30186 |
| 581 | 0.26439 | 0.09534 | 0.21229 | 0.50374 | 0.68075 | 0.03153 | 0.01159 | 0.14044 | 0.30262 |
| 580 | 0.26806 | 0.09795 | 0.21418 | 0.50674 | 0.6798 | 0.03189 | 0.01122 | 0.14305 | 0.30938 |
| 579 | 0.27683 | 0.09939 | 0.21683 | 0.50644 | 0.67978 | 0.03155 | 0.01094 | 0.14657 | 0.31159 |
| 578 | 0.2833 | 0.10024 | 0.21937 | 0.50816 | 0.67954 | 0.03215 | 0.01098 | 0.14909 | 0.31713 |
| 577 | 0.28998 | 0.10153 | 0.22051 | 0.51062 | 0.67984 | 0.03233 | 0.01121 | 0.15328 | 0.32152 |
| 576 | 0.29811 | 0.10257 | 0.2244 | 0.50917 | 0.67963 | 0.03236 | 0.01108 | 0.15643 | 0.3252 |
| 575 | 0.30343 | 0.10582 | 0.22674 | 0.51609 | 0.67927 | 0.03174 | 0.01087 | 0.15945 | 0.3309 |
| 574 | 0.31266 | 0.10686 | 0.22908 | 0.51375 | 0.67961 | 0.0316 | 0.01128 | 0.16446 | 0.33539 |
| 573 | 0.3201 | 0.10711 | 0.23202 | 0.51961 | 0.68018 | 0.03263 | 0.01143 | 0.16763 | 0.34151 |
| 572 | 0.32801 | 0.10843 | 0.23439 | 0.51986 | 0.68082 | 0.03197 | 0.01062 | 0.17089 | 0.34459 |
| 571 | 0.33411 | 0.11086 | 0.2368 | 0.52203 | 0.68113 | 0.03232 | 0.0114 | 0.17586 | 0.34917 |
| 570 | 0.34445 | 0.11364 | 0.24014 | 0.52334 | 0.68175 | 0.03259 | 0.01111 | 0.17928 | 0.35561 |
| 569 | 0.35306 | 0.11482 | 0.24327 | 0.52781 | 0.68198 | 0.03201 | 0.01113 | 0.18505 | 0.3586 |
| 568 | 0.36296 | 0.11561 | 0.24596 | 0.52899 | 0.6827 | 0.03299 | 0.011 | 0.18675 | 0.36714 |
| 567 | 0.37128 | 0.1184 | 0.24873 | 0.53041 | 0.68394 | 0.03299 | 0.01087 | 0.189 | 0.37074 |
| 566 | 0.38084 | 0.11881 | 0.2516 | 0.53565 | 0.68477 | 0.03309 | 0.01158 | 0.19579 | 0.3742 |
| 565 | 0.3904 | 0.1205 | 0.25537 | 0.53692 | 0.68579 | 0.03319 | 0.01106 | 0.19563 | 0.38196 |
| 564 | 0.40027 | 0.12314 | 0.25833 | 0.54221 | 0.68635 | 0.03295 | 0.01152 | 0.20345 | 0.38655 |
| 563 | 0.40961 | 0.12296 | 0.26237 | 0.54313 | 0.68815 | 0.03354 | 0.01088 | 0.205 | 0.39207 |
| 562 | 0.4206 | 0.12765 | 0.26366 | 0.54648 | 0.68979 | 0.03333 | 0.01182 | 0.20863 | 0.3974 |
| 561 | 0.43058 | 0.12752 | 0.26733 | 0.55068 | 0.69084 | 0.03332 | 0.01137 | 0.21335 | 0.40126 |
| 560 | 0.44169 | 0.12973 | 0.27048 | 0.55397 | 0.69203 | 0.0335 | 0.01158 | 0.2162 | 0.40742 |
| 559 | 0.45472 | 0.13198 | 0.27475 | 0.55652 | 0.6938 | 0.03361 | 0.01159 | 0.21947 | 0.4139 |
| 558 | 0.46285 | 0.13261 | 0.27666 | 0.55867 | 0.69556 | 0.03376 | 0.01143 | 0.22253 | 0.42037 |
| 557 | 0.47448 | 0.13594 | 0.27957 | 0.56226 | 0.69693 | 0.03352 | 0.01146 | 0.22671 | 0.42382 |
| 556 | 0.4863 | 0.1376 | 0.28377 | 0.56681 | 0.69836 | 0.03382 | 0.01112 | 0.23196 | 0.42939 |
| 555 | 0.49946 | 0.14008 | 0.2856 | 0.57058 | 0.70056 | 0.034 | 0.01134 | 0.23487 | 0.43562 |
| 554 | 0.50995 | 0.14116 | 0.28847 | 0.57559 | 0.70223 | 0.03447 | 0.01162 | 0.23857 | 0.44123 |
| 553 | 0.52242 | 0.14168 | 0.2913 | 0.57763 | 0.70419 | 0.03369 | 0.01139 | 0.2427 | 0.44579 |
| 552 | 0.53512 | 0.14586 | 0.29663 | 0.58264 | 0.70562 | 0.03407 | 0.01104 | 0.24332 | 0.44873 |
| 551 | 0.54645 | 0.14628 | 0.29691 | 0.58653 | 0.70792 | 0.03424 | 0.01164 | 0.2486 | 0.45354 |
| 550 | 0.5598 | 0.14883 | 0.30209 | 0.59143 | 0.70962 | 0.03392 | 0.01155 | 0.25168 | 0.45825 |
| 549 | 0.57118 | 0.1505 | 0.30472 | 0.59629 | 0.71244 | 0.03454 | 0.01161 | 0.25349 | 0.46072 |
| 548 | 0.58393 | 0.15377 | 0.3085 | 0.59643 | 0.71447 | 0.03471 | 0.01187 | 0.25616 | 0.46604 |
| 547 | 0.5975 | 0.15547 | 0.31119 | 0.60355 | 0.71601 | 0.03456 | 0.01175 | 0.25993 | 0.46945 |
| 546 | 0.60915 | 0.15614 | 0.31458 | 0.60606 | 0.71793 | 0.03448 | 0.01179 | 0.26245 | 0.47481 |
| 545 | 0.62274 | 0.15854 | 0.31706 | 0.60875 | 0.72011 | 0.03476 | 0.01167 | 0.26463 | 0.47903 |
| 544 | 0.63446 | 0.16235 | 0.31959 | 0.61516 | 0.72202 | 0.03497 | 0.0122 | 0.26427 | 0.48126 |
| 543 | 0.64592 | 0.16359 | 0.32303 | 0.61554 | 0.72357 | 0.03459 | 0.01217 | 0.26737 | 0.48489 |
| 542 | 0.65844 | 0.16358 | 0.32436 | 0.62111 | 0.72566 | 0.03489 | 0.01208 | 0.26796 | 0.48608 |
| 541 | 0.6702 | 0.16675 | 0.32756 | 0.62491 | 0.72722 | 0.03495 | 0.01174 | 0.27034 | 0.48761 |
| 540 | 0.68171 | 0.16829 | 0.3307 | 0.6271 | 0.72937 | 0.03527 | 0.01234 | 0.27114 | 0.49168 |
| 539 | 0.69375 | 0.16941 | 0.33373 | 0.63321 | 0.73022 | 0.03492 | 0.0117 | 0.27178 | 0.49366 |
| 538 | 0.70495 | 0.17167 | 0.33251 | 0.63373 | 0.7317 | 0.03565 | 0.01235 | 0.27416 | 0.49448 |
| 537 | 0.71477 | 0.17327 | 0.33713 | 0.63586 | 0.73315 | 0.03503 | 0.01211 | 0.27273 | 0.49498 |
| 536 | 0.72577 | 0.17397 | 0.33836 | 0.64018 | 0.73409 | 0.03554 | 0.01224 | 0.27498 | 0.49615 |
| 535 | 0.73652 | 0.17487 | 0.33971 | 0.64118 | 0.73539 | 0.03477 | 0.01157 | 0.27517 | 0.49556 |
| 534 | 0.74755 | 0.17918 | 0.34221 | 0.64876 | 0.73617 | 0.03501 | 0.01191 | 0.27381 | 0.49796 |
| 533 | 0.75536 | 0.17888 | 0.34359 | 0.64742 | 0.73752 | 0.03521 | 0.01175 | 0.27169 | 0.49763 |
| 532 | 0.7634 | 0.17967 | 0.34435 | 0.65352 | 0.73742 | 0.0351 | 0.0123 | 0.2714 | 0.49499 |
| 531 | 0.77291 | 0.18248 | 0.34619 | 0.65426 | 0.73819 | 0.03526 | 0.01216 | 0.27137 | 0.49449 |
| 530 | 0.77877 | 0.18239 | 0.34796 | 0.65268 | 0.73819 | 0.03497 | 0.01153 | 0.27093 | 0.49195 |
| 529 | 0.78745 | 0.18228 | 0.34806 | 0.65598 | 0.7389 | 0.03522 | 0.01227 | 0.26751 | 0.49108 |
| 528 | 0.79116 | 0.18313 | 0.34889 | 0.6563 | 0.73859 | 0.03509 | 0.01231 | 0.26848 | 0.48893 |
| 527 | 0.79776 | 0.18268 | 0.34978 | 0.65925 | 0.73776 | 0.03538 | 0.012 | 0.26464 | 0.48677 |
| 526 | 0.80379 | 0.18471 | 0.35003 | 0.65745 | 0.7371 | 0.03488 | 0.01249 | 0.26526 | 0.48272 |
| 525 | 0.80814 | 0.18493 | 0.35082 | 0.66063 | 0.7366 | 0.03531 | 0.01255 | 0.26123 | 0.48187 |
| 524 | 0.81154 | 0.18375 | 0.35027 | 0.65832 | 0.73482 | 0.03445 | 0.01162 | 0.26185 | 0.47872 |
| 523 | 0.81506 | 0.18506 | 0.35133 | 0.66041 | 0.73371 | 0.03444 | 0.01231 | 0.26025 | 0.47315 |
| 522 | 0.81895 | 0.18393 | 0.34995 | 0.65542 | 0.7323 | 0.03477 | 0.01205 | 0.25383 | 0.47092 |
| 521 | 0.81762 | 0.18482 | 0.35119 | 0.65817 | 0.73128 | 0.03537 | 0.01261 | 0.25445 | 0.46757 |
| 520 | 0.82102 | 0.18571 | 0.35091 | 0.65272 | 0.72817 | 0.03514 | 0.01172 | 0.2499 | 0.4627 |
| 519 | 0.82285 | 0.18345 | 0.3503 | 0.65089 | 0.72648 | 0.03469 | 0.0122 | 0.24793 | 0.45847 |
| 518 | 0.82092 | 0.18541 | 0.3489 | 0.6536 | 0.72352 | 0.03451 | 0.01197 | 0.24768 | 0.45478 |
| 517 | 0.82024 | 0.18341 | 0.34855 | 0.64808 | 0.72021 | 0.03419 | 0.01175 | 0.24255 | 0.4497 |
| 516 | 0.81885 | 0.18502 | 0.34866 | 0.64506 | 0.71766 | 0.03415 | 0.01245 | 0.24072 | 0.44444 |
| 515 | 0.81713 | 0.18364 | 0.34673 | 0.64192 | 0.71359 | 0.03412 | 0.01202 | 0.23703 | 0.44024 |
| 514 | 0.81393 | 0.18103 | 0.3442 | 0.64008 | 0.71006 | 0.03371 | 0.01231 | 0.23354 | 0.43609 |
| 513 | 0.81028 | 0.18014 | 0.34284 | 0.63602 | 0.70664 | 0.03356 | 0.01231 | 0.23116 | 0.42971 |
| 512 | 0.80685 | 0.18218 | 0.34336 | 0.6321 | 0.7015 | 0.0336 | 0.01229 | 0.22854 | 0.42541 |
| 511 | 0.80406 | 0.18009 | 0.34118 | 0.62517 | 0.6975 | 0.03321 | 0.01188 | 0.225 | 0.42075 |
| 510 | 0.79739 | 0.17838 | 0.33829 | 0.62318 | 0.6926 | 0.03316 | 0.01164 | 0.22226 | 0.4146 |
| 509 | 0.79423 | 0.17591 | 0.33837 | 0.61769 | 0.68831 | 0.03305 | 0.01212 | 0.21571 | 0.40946 |
| 508 | 0.78775 | 0.17609 | 0.33588 | 0.614 | 0.68399 | 0.03317 | 0.01226 | 0.21491 | 0.40616 |
| 507 | 0.78058 | 0.1743 | 0.33344 | 0.60938 | 0.6785 | 0.03313 | 0.01234 | 0.21267 | 0.39973 |
| 506 | 0.77581 | 0.1743 | 0.33226 | 0.6014 | 0.67312 | 0.0331 | 0.01204 | 0.20844 | 0.39497 |
| 505 | 0.76897 | 0.17081 | 0.33103 | 0.59614 | 0.66814 | 0.03266 | 0.01237 | 0.20595 | 0.38979 |
| 504 | 0.76129 | 0.16841 | 0.3271 | 0.59167 | 0.66263 | 0.03283 | 0.01231 | 0.20272 | 0.38585 |
| 503 | 0.75576 | 0.16903 | 0.32758 | 0.58555 | 0.65756 | 0.03251 | 0.01231 | 0.20094 | 0.37873 |
| 502 | 0.74896 | 0.16735 | 0.32297 | 0.57811 | 0.65216 | 0.03212 | 0.01232 | 0.19589 | 0.3753 |
| 501 | 0.74064 | 0.16682 | 0.32112 | 0.57389 | 0.64654 | 0.03222 | 0.01214 | 0.193 | 0.37064 |
| 500 | 0.73502 | 0.1625 | 0.31974 | 0.56518 | 0.64132 | 0.0322 | 0.01227 | 0.19163 | 0.36601 |
| 499 | 0.72865 | 0.16353 | 0.31799 | 0.56024 | 0.6358 | 0.03191 | 0.01193 | 0.18856 | 0.36157 |
| 498 | 0.71988 | 0.16134 | 0.31546 | 0.55253 | 0.63062 | 0.03174 | 0.01175 | 0.18525 | 0.35668 |
| 497 | 0.71319 | 0.16054 | 0.31355 | 0.55046 | 0.62582 | 0.03183 | 0.01217 | 0.1835 | 0.35214 |
| 496 | 0.70377 | 0.15768 | 0.31075 | 0.54415 | 0.62027 | 0.03104 | 0.01165 | 0.18005 | 0.34873 |
| 495 | 0.69785 | 0.15638 | 0.30891 | 0.5358 | 0.61581 | 0.03134 | 0.01195 | 0.17941 | 0.34486 |
| 494 | 0.69263 | 0.15404 | 0.30835 | 0.5303 | 0.61125 | 0.0316 | 0.01195 | 0.17521 | 0.34255 |
| 493 | 0.68431 | 0.1531 | 0.3045 | 0.52866 | 0.60635 | 0.03098 | 0.01158 | 0.17585 | 0.33707 |
| 492 | 0.67687 | 0.15287 | 0.30399 | 0.51859 | 0.60177 | 0.03074 | 0.01153 | 0.1705 | 0.33195 |
| 491 | 0.66893 | 0.15162 | 0.30174 | 0.51703 | 0.59721 | 0.03077 | 0.01141 | 0.17043 | 0.33025 |
| 490 | 0.66433 | 0.14936 | 0.2989 | 0.51015 | 0.59418 | 0.03131 | 0.01184 | 0.16715 | 0.3272 |
| 489 | 0.6566 | 0.14789 | 0.2987 | 0.5071 | 0.59005 | 0.03042 | 0.01144 | 0.16642 | 0.32429 |
| 488 | 0.6495 | 0.14444 | 0.29529 | 0.50174 | 0.58642 | 0.03034 | 0.01187 | 0.16304 | 0.32207 |
| 487 | 0.64447 | 0.14401 | 0.2936 | 0.49647 | 0.5827 | 0.03021 | 0.01179 | 0.16161 | 0.31777 |
| 486 | 0.63865 | 0.14419 | 0.29354 | 0.49301 | 0.57959 | 0.03073 | 0.01179 | 0.16242 | 0.3164 |
| 485 | 0.6331 | 0.14336 | 0.29232 | 0.49106 | 0.57637 | 0.03024 | 0.01178 | 0.15831 | 0.31517 |
| 484 | 0.62667 | 0.14174 | 0.29097 | 0.4888 | 0.57324 | 0.0305 | 0.01184 | 0.16068 | 0.31142 |
| 483 | 0.6221 | 0.14153 | 0.2885 | 0.48024 | 0.57037 | 0.03009 | 0.01141 | 0.1577 | 0.31033 |
| 482 | 0.61674 | 0.14086 | 0.28754 | 0.47835 | 0.56974 | 0.03069 | 0.01201 | 0.15522 | 0.30989 |
| 481 | 0.61148 | 0.14017 | 0.28613 | 0.475 | 0.56657 | 0.03027 | 0.01214 | 0.15648 | 0.30634 |
| 480 | 0.60515 | 0.13912 | 0.28511 | 0.47573 | 0.56348 | 0.03021 | 0.01157 | 0.15478 | 0.30468 |
| 479 | 0.60287 | 0.13943 | 0.28423 | 0.46683 | 0.56287 | 0.03061 | 0.01194 | 0.1541 | 0.30162 |
| 478 | 0.59812 | 0.13734 | 0.28289 | 0.46898 | 0.56075 | 0.03022 | 0.01241 | 0.15131 | 0.30093 |
| 477 | 0.59322 | 0.13751 | 0.28169 | 0.46464 | 0.55854 | 0.03017 | 0.012 | 0.15152 | 0.30025 |
| 476 | 0.59003 | 0.13657 | 0.27987 | 0.46287 | 0.55719 | 0.02997 | 0.01198 | 0.15194 | 0.30084 |
| 475 | 0.58686 | 0.13733 | 0.28103 | 0.46295 | 0.55621 | 0.03043 | 0.01177 | 0.14829 | 0.29605 |
| 474 | 0.58273 | 0.13562 | 0.27948 | 0.4583 | 0.55518 | 0.03086 | 0.01222 | 0.15039 | 0.29874 |
| 473 | 0.57904 | 0.1347 | 0.27807 | 0.4541 | 0.55397 | 0.03056 | 0.01229 | 0.14815 | 0.29636 |
| 472 | 0.5747 | 0.13489 | 0.27757 | 0.45469 | 0.55284 | 0.03026 | 0.01181 | 0.14863 | 0.29469 |
| 471 | 0.5725 | 0.1341 | 0.27716 | 0.45291 | 0.55219 | 0.03027 | 0.01225 | 0.14665 | 0.29425 |
| 470 | 0.57017 | 0.13496 | 0.27628 | 0.45215 | 0.55202 | 0.03077 | 0.01211 | 0.14797 | 0.29273 |
| 469 | 0.566 | 0.13253 | 0.27305 | 0.44826 | 0.5507 | 0.03009 | 0.01182 | 0.14668 | 0.29239 |
| 468 | 0.56443 | 0.13398 | 0.27541 | 0.44865 | 0.55048 | 0.0306 | 0.01189 | 0.14414 | 0.29296 |
| 467 | 0.56047 | 0.13386 | 0.2745 | 0.45009 | 0.54985 | 0.03073 | 0.01259 | 0.14409 | 0.29057 |
| 466 | 0.55814 | 0.13376 | 0.27506 | 0.44557 | 0.54957 | 0.03054 | 0.01274 | 0.14458 | 0.29009 |
| 465 | 0.55594 | 0.1319 | 0.27537 | 0.44717 | 0.54939 | 0.03047 | 0.01222 | 0.14346 | 0.29027 |
| 464 | 0.5559 | 0.13492 | 0.27458 | 0.44793 | 0.54956 | 0.03037 | 0.01218 | 0.14463 | 0.28918 |
| 463 | 0.55141 | 0.13156 | 0.27141 | 0.44692 | 0.54898 | 0.03074 | 0.01245 | 0.14371 | 0.28872 |
| 462 | 0.55021 | 0.13334 | 0.27183 | 0.44153 | 0.5485 | 0.03024 | 0.01216 | 0.14279 | 0.28936 |
| 461 | 0.54904 | 0.13276 | 0.27144 | 0.44549 | 0.54926 | 0.03095 | 0.01226 | 0.14443 | 0.28848 |
| 460 | 0.54731 | 0.13335 | 0.27137 | 0.44073 | 0.54858 | 0.03051 | 0.01245 | 0.14413 | 0.28767 |
| 459 | 0.54583 | 0.13204 | 0.27116 | 0.44227 | 0.5492 | 0.0302 | 0.01254 | 0.14008 | 0.28871 |
| 458 | 0.54386 | 0.13262 | 0.27154 | 0.4373 | 0.54945 | 0.03104 | 0.0128 | 0.14342 | 0.28765 |
| 457 | 0.54345 | 0.13267 | 0.27105 | 0.44239 | 0.54882 | 0.03069 | 0.01213 | 0.14327 | 0.28704 |
| 456 | 0.54101 | 0.13223 | 0.27033 | 0.44101 | 0.54959 | 0.03069 | 0.01241 | 0.14405 | 0.28907 |
| 455 | 0.54046 | 0.13425 | 0.27098 | 0.43985 | 0.55019 | 0.03052 | 0.01227 | 0.143 | 0.28642 |
| 454 | 0.53852 | 0.13102 | 0.26896 | 0.44143 | 0.54997 | 0.03027 | 0.01196 | 0.14271 | 0.28902 |
| 453 | 0.53764 | 0.13356 | 0.27153 | 0.44046 | 0.55098 | 0.03077 | 0.01228 | 0.14189 | 0.28817 |
| 452 | 0.53682 | 0.13372 | 0.27146 | 0.44082 | 0.55102 | 0.03108 | 0.01249 | 0.14187 | 0.28591 |
| 451 | 0.5359 | 0.13419 | 0.27045 | 0.43869 | 0.55232 | 0.03143 | 0.01264 | 0.14454 | 0.29012 |
| 450 | 0.53596 | 0.13447 | 0.27124 | 0.43959 | 0.5528 | 0.03099 | 0.01279 | 0.14079 | 0.28756 |
| 449 | 0.53501 | 0.13406 | 0.27121 | 0.4394 | 0.55289 | 0.03118 | 0.01314 | 0.1438 | 0.28827 |
| 448 | 0.53529 | 0.13348 | 0.26997 | 0.4419 | 0.55351 | 0.03152 | 0.01291 | 0.1445 | 0.28952 |
| 447 | 0.53426 | 0.13399 | 0.27128 | 0.43937 | 0.55453 | 0.03173 | 0.01302 | 0.14327 | 0.29112 |
| 446 | 0.53169 | 0.13478 | 0.27161 | 0.4436 | 0.55449 | 0.03162 | 0.01328 | 0.13986 | 0.2888 |
| 445 | 0.53202 | 0.13352 | 0.27141 | 0.43998 | 0.55601 | 0.03221 | 0.01332 | 0.14133 | 0.29005 |
| 444 | 0.53091 | 0.13406 | 0.27138 | 0.44207 | 0.55603 | 0.03129 | 0.01328 | 0.14184 | 0.28943 |
| 443 | 0.52936 | 0.13464 | 0.27033 | 0.44066 | 0.55699 | 0.03194 | 0.01307 | 0.14345 | 0.28987 |
| 442 | 0.53072 | 0.13576 | 0.27162 | 0.44178 | 0.55766 | 0.03174 | 0.01339 | 0.14313 | 0.29109 |
| 441 | 0.53061 | 0.13508 | 0.27097 | 0.44064 | 0.55789 | 0.0318 | 0.0131 | 0.14182 | 0.2908 |
| 440 | 0.53077 | 0.1357 | 0.27127 | 0.44182 | 0.55803 | 0.03142 | 0.01248 | 0.14095 | 0.29176 |
| 439 | 0.53043 | 0.13684 | 0.27258 | 0.44184 | 0.55906 | 0.03209 | 0.01338 | 0.14442 | 0.29152 |
| 438 | 0.53133 | 0.13696 | 0.27162 | 0.44069 | 0.5608 | 0.03201 | 0.01303 | 0.14401 | 0.29306 |
| 437 | 0.53087 | 0.13708 | 0.27133 | 0.44417 | 0.56094 | 0.03241 | 0.01334 | 0.14287 | 0.29214 |
| 436 | 0.53156 | 0.13699 | 0.27288 | 0.44352 | 0.5617 | 0.03208 | 0.0134 | 0.14424 | 0.29343 |
| 435 | 0.53135 | 0.13597 | 0.27489 | 0.44252 | 0.56282 | 0.03215 | 0.01297 | 0.14362 | 0.29468 |
| 434 | 0.53089 | 0.13929 | 0.27402 | 0.44354 | 0.56376 | 0.0323 | 0.01332 | 0.14335 | 0.29549 |
| 433 | 0.52986 | 0.13822 | 0.27355 | 0.44331 | 0.56434 | 0.03253 | 0.01366 | 0.14525 | 0.2952 |
| 432 | 0.53026 | 0.13929 | 0.27293 | 0.44576 | 0.56557 | 0.03229 | 0.01354 | 0.14387 | 0.29612 |
| 431 | 0.52971 | 0.13835 | 0.27346 | 0.44183 | 0.56626 | 0.03215 | 0.01358 | 0.14411 | 0.2949 |
| 430 | 0.53181 | 0.13883 | 0.27338 | 0.44544 | 0.56726 | 0.03274 | 0.01338 | 0.145 | 0.29554 |
| 429 | 0.53154 | 0.14028 | 0.27419 | 0.44714 | 0.56815 | 0.03293 | 0.01356 | 0.14551 | 0.29439 |
| 428 | 0.53224 | 0.14018 | 0.27462 | 0.44609 | 0.56915 | 0.03232 | 0.01386 | 0.14404 | 0.29712 |
| 427 | 0.53051 | 0.13827 | 0.27706 | 0.44721 | 0.57045 | 0.03343 | 0.01379 | 0.14586 | 0.29616 |
| 426 | 0.53217 | 0.14016 | 0.27619 | 0.44938 | 0.5708 | 0.03346 | 0.01423 | 0.14444 | 0.29575 |
| 425 | 0.53333 | 0.14067 | 0.27592 | 0.44677 | 0.57195 | 0.03294 | 0.01401 | 0.14523 | 0.29916 |
| 424 | 0.53238 | 0.14086 | 0.27596 | 0.45013 | 0.57343 | 0.03354 | 0.01399 | 0.14621 | 0.29844 |
| 423 | 0.53395 | 0.14164 | 0.27687 | 0.45019 | 0.57386 | 0.03315 | 0.01433 | 0.14497 | 0.29796 |
| 422 | 0.53457 | 0.142 | 0.27714 | 0.44913 | 0.57529 | 0.03353 | 0.01403 | 0.14503 | 0.29893 |
| 421 | 0.53459 | 0.14196 | 0.2775 | 0.45584 | 0.57597 | 0.03387 | 0.01406 | 0.14636 | 0.30051 |
| 420 | 0.5343 | 0.14188 | 0.27739 | 0.45015 | 0.57772 | 0.03392 | 0.0141 | 0.14766 | 0.30096 |
| 419 | 0.53826 | 0.14346 | 0.27823 | 0.45482 | 0.57809 | 0.03366 | 0.01353 | 0.14714 | 0.30073 |
| 418 | 0.53552 | 0.1437 | 0.27783 | 0.45403 | 0.57934 | 0.03353 | 0.01426 | 0.14815 | 0.30136 |
| 417 | 0.53827 | 0.14363 | 0.27989 | 0.45502 | 0.58019 | 0.03402 | 0.01409 | 0.14597 | 0.30194 |
| 416 | 0.53904 | 0.14485 | 0.27973 | 0.4538 | 0.58095 | 0.03405 | 0.01415 | 0.15043 | 0.30309 |
| 415 | 0.53845 | 0.14493 | 0.27944 | 0.45545 | 0.58223 | 0.03383 | 0.01422 | 0.14691 | 0.30339 |
| 414 | 0.54028 | 0.14721 | 0.28149 | 0.45771 | 0.584 | 0.03458 | 0.01447 | 0.14771 | 0.30419 |
| 413 | 0.54096 | 0.14613 | 0.28095 | 0.45944 | 0.58453 | 0.03415 | 0.01433 | 0.14711 | 0.30437 |
| 412 | 0.54062 | 0.14494 | 0.28023 | 0.45729 | 0.5854 | 0.0341 | 0.01456 | 0.14909 | 0.30574 |
| 411 | 0.54148 | 0.1471 | 0.28167 | 0.46069 | 0.58667 | 0.03455 | 0.01425 | 0.14956 | 0.30618 |
| 410 | 0.54208 | 0.14776 | 0.28269 | 0.46136 | 0.58823 | 0.03438 | 0.0146 | 0.15001 | 0.30526 |
| 409 | 0.54286 | 0.14844 | 0.28285 | 0.46003 | 0.58941 | 0.03524 | 0.0151 | 0.14923 | 0.30843 |
| 408 | 0.54301 | 0.14798 | 0.28391 | 0.46492 | 0.59013 | 0.0341 | 0.01446 | 0.15121 | 0.30781 |
| 407 | 0.5461 | 0.15218 | 0.28285 | 0.46281 | 0.59158 | 0.03478 | 0.01464 | 0.1525 | 0.30842 |
| 406 | 0.54468 | 0.14956 | 0.28235 | 0.46466 | 0.59297 | 0.03474 | 0.01478 | 0.15002 | 0.31052 |
| 405 | 0.54623 | 0.14999 | 0.28527 | 0.46199 | 0.59381 | 0.03523 | 0.01468 | 0.15162 | 0.3098 |
| 404 | 0.54775 | 0.14932 | 0.28426 | 0.46511 | 0.59508 | 0.03462 | 0.01431 | 0.15308 | 0.31058 |
| 403 | 0.54908 | 0.15169 | 0.28628 | 0.46838 | 0.59593 | 0.0353 | 0.01461 | 0.15125 | 0.31237 |
| 402 | 0.55152 | 0.15083 | 0.28677 | 0.4671 | 0.59762 | 0.03544 | 0.01459 | 0.15297 | 0.31181 |
| 401 | 0.55263 | 0.1516 | 0.28584 | 0.46997 | 0.59885 | 0.03542 | 0.01534 | 0.15312 | 0.31286 |
| 400 | 0.55244 | 0.15344 | 0.28626 | 0.46758 | 0.59981 | 0.03538 | 0.0146 | 0.15238 | 0.31279 |
| 399 | 0.55479 | 0.1546 | 0.28802 | 0.46903 | 0.60115 | 0.03584 | 0.01488 | 0.1553 | 0.31414 |
| 398 | 0.5577 | 0.15722 | 0.28937 | 0.47361 | 0.6021 | 0.03579 | 0.015 | 0.15348 | 0.31273 |
| 397 | 0.55736 | 0.15544 | 0.28917 | 0.47256 | 0.6036 | 0.03562 | 0.01491 | 0.15454 | 0.31557 |
| 396 | 0.55965 | 0.15531 | 0.29203 | 0.47375 | 0.60537 | 0.03597 | 0.01558 | 0.15451 | 0.31598 |
| 395 | 0.56067 | 0.15783 | 0.29017 | 0.47491 | 0.60665 | 0.03627 | 0.01484 | 0.15578 | 0.31736 |
| 394 | 0.56262 | 0.15824 | 0.29217 | 0.47436 | 0.6079 | 0.03645 | 0.0157 | 0.15647 | 0.31693 |
| 393 | 0.56417 | 0.15831 | 0.29188 | 0.47951 | 0.60866 | 0.03622 | 0.01532 | 0.15575 | 0.31808 |
| 392 | 0.56368 | 0.15921 | 0.29335 | 0.48105 | 0.61029 | 0.03639 | 0.01573 | 0.15574 | 0.31852 |
| 391 | 0.56657 | 0.15963 | 0.29549 | 0.47965 | 0.6116 | 0.03687 | 0.01504 | 0.15618 | 0.32105 |
| 390 | 0.56832 | 0.16054 | 0.29541 | 0.48253 | 0.61293 | 0.03693 | 0.01658 | 0.16085 | 0.32217 |
| 389 | 0.57029 | 0.16242 | 0.29603 | 0.48401 | 0.61413 | 0.0368 | 0.01614 | 0.15531 | 0.32135 |
| 388 | 0.57237 | 0.16208 | 0.29614 | 0.4821 | 0.61446 | 0.03676 | 0.01544 | 0.15891 | 0.3236 |
| 387 | 0.57265 | 0.16129 | 0.29709 | 0.48345 | 0.61662 | 0.03694 | 0.01566 | 0.15717 | 0.32352 |
| 386 | 0.57383 | 0.16328 | 0.29722 | 0.4849 | 0.61703 | 0.03728 | 0.01561 | 0.15843 | 0.32465 |
| 385 | 0.57735 | 0.16483 | 0.29831 | 0.48383 | 0.61885 | 0.03708 | 0.01597 | 0.15791 | 0.32469 |
| 384 | 0.57867 | 0.16526 | 0.29927 | 0.48601 | 0.62025 | 0.03758 | 0.01647 | 0.15786 | 0.32649 |
| 383 | 0.57815 | 0.16457 | 0.29984 | 0.48739 | 0.62083 | 0.03717 | 0.01594 | 0.15926 | 0.32604 |
| 382 | 0.58247 | 0.16804 | 0.29972 | 0.48474 | 0.62246 | 0.03756 | 0.01602 | 0.16111 | 0.32624 |
| 381 | 0.5835 | 0.16714 | 0.30289 | 0.49381 | 0.62388 | 0.03804 | 0.01589 | 0.15893 | 0.32759 |
| 380 | 0.58503 | 0.16923 | 0.30201 | 0.49044 | 0.62418 | 0.03718 | 0.01589 | 0.15949 | 0.32807 |
| 379 | 0.58577 | 0.16799 | 0.30224 | 0.49297 | 0.62534 | 0.03794 | 0.01589 | 0.1594 | 0.32977 |
| 378 | 0.58773 | 0.16877 | 0.30265 | 0.49357 | 0.62686 | 0.03808 | 0.01591 | 0.16273 | 0.33018 |
| 377 | 0.59008 | 0.17176 | 0.30572 | 0.49592 | 0.62902 | 0.03955 | 0.01733 | 0.16145 | 0.33215 |
| 376 | 0.58987 | 0.17152 | 0.3045 | 0.49464 | 0.62913 | 0.03874 | 0.01669 | 0.16047 | 0.33212 |
| 375 | 0.59296 | 0.17028 | 0.30533 | 0.49756 | 0.62925 | 0.03718 | 0.01601 | 0.16565 | 0.33272 |
| 374 | 0.59436 | 0.17211 | 0.30557 | 0.49646 | 0.63229 | 0.03854 | 0.01689 | 0.16004 | 0.33238 |
| 373 | 0.59591 | 0.17512 | 0.30851 | 0.50101 | 0.63297 | 0.03831 | 0.01633 | 0.15988 | 0.3338 |
| 372 | 0.59979 | 0.17434 | 0.3102 | 0.50005 | 0.63349 | 0.03872 | 0.01685 | 0.16181 | 0.33431 |
| 371 | 0.59943 | 0.17448 | 0.30882 | 0.49861 | 0.63452 | 0.03964 | 0.01759 | 0.16347 | 0.33439 |
| 370 | 0.60152 | 0.17613 | 0.30993 | 0.50699 | 0.63564 | 0.03908 | 0.01685 | 0.16185 | 0.33421 |
| 369 | 0.60452 | 0.17751 | 0.3116 | 0.49965 | 0.63595 | 0.04039 | 0.01717 | 0.16033 | 0.33689 |
| 368 | 0.61 | 0.1888 | 0.31575 | 0.49822 | 0.63766 | 0.03945 | 0.01706 | 0.16163 | 0.33859 |
| 367 | 0.61402 | 0.18781 | 0.31536 | 0.50296 | 0.6377 | 0.03989 | 0.01687 | 0.16076 | 0.33739 |
| 366 | 0.61275 | 0.18735 | 0.31665 | 0.50339 | 0.63895 | 0.03886 | 0.01685 | 0.16466 | 0.33915 |
| 365 | 0.61483 | 0.18875 | 0.31595 | 0.50546 | 0.64021 | 0.0397 | 0.01797 | 0.16374 | 0.33942 |
| 364 | 0.61734 | 0.18964 | 0.31903 | 0.50269 | 0.6412 | 0.03987 | 0.01712 | 0.1615 | 0.33884 |
| 363 | 0.6166 | 0.19006 | 0.31874 | 0.50011 | 0.64151 | 0.04026 | 0.01755 | 0.16211 | 0.34079 |
| 362 | 0.62088 | 0.19265 | 0.31949 | 0.504 | 0.64317 | 0.0408 | 0.01766 | 0.16095 | 0.34242 |
| 361 | 0.61982 | 0.19107 | 0.32086 | 0.50551 | 0.64127 | 0.03924 | 0.01842 | 0.16426 | 0.34122 |
| 360 | 0.61976 | 0.19193 | 0.32069 | 0.50158 | 0.64406 | 0.04141 | 0.01755 | 0.15914 | 0.34139 |
| 359 | 0.61841 | 0.1931 | 0.32038 | 0.5002 | 0.6451 | 0.04126 | 0.01731 | 0.16274 | 0.34377 |
| 358 | 0.61886 | 0.19386 | 0.32108 | 0.49847 | 0.64439 | 0.03919 | 0.01711 | 0.16513 | 0.3439 |
| 357 | 0.62034 | 0.19622 | 0.32491 | 0.49772 | 0.64643 | 0.04306 | 0.01809 | 0.1619 | 0.34505 |
| 356 | 0.61589 | 0.19263 | 0.32127 | 0.4971 | 0.64361 | 0.03847 | 0.01801 | 0.15859 | 0.34561 |
| 355 | 0.61853 | 0.19498 | 0.32288 | 0.50091 | 0.64683 | 0.04069 | 0.01699 | 0.15973 | 0.34217 |
| 354 | 0.61175 | 0.19151 | 0.32052 | 0.49723 | 0.64723 | 0.04164 | 0.01728 | 0.1521 | 0.34552 |
| 353 | 0.60991 | 0.19401 | 0.32007 | 0.48693 | 0.64878 | 0.04313 | 0.01836 | 0.1554 | 0.34343 |
| 352 | 0.60604 | 0.19459 | 0.31971 | 0.47831 | 0.64921 | 0.04218 | 0.02153 | 0.15496 | 0.34443 |
| 351 | 0.59222 | 0.18884 | 0.31709 | 0.47273 | 0.65135 | 0.04215 | 0.01921 | 0.14564 | 0.34017 |
| 350 | 0.58723 | 0.18869 | 0.3119 | 0.47182 | 0.64648 | 0.03974 | 0.01633 | 0.14593 | 0.34148 |
| 349 | 0.57654 | 0.18708 | 0.31211 | 0.46149 | 0.64887 | 0.04359 | 0.0205 | 0.14087 | 0.33964 |
| 348 | 0.45125 | -0.03565 | 0.29893 | 0.51977 | 0.65048 | 0.03719 | 0.01824 | 0.17478 | 0.35631 |
| 347 | 0.45242 | -0.03493 | 0.29985 | 0.52068 | 0.65163 | 0.03799 | 0.01834 | 0.1744 | 0.35723 |
| 346 | 0.45361 | -0.03527 | 0.29954 | 0.52177 | 0.6528 | 0.03705 | 0.0183 | 0.17544 | 0.35819 |
| 345 | 0.45537 | -0.03418 | 0.30055 | 0.52241 | 0.65323 | 0.03728 | 0.01813 | 0.17473 | 0.35852 |
| 344 | 0.45623 | -0.03368 | 0.3007 | 0.524 | 0.65529 | 0.03801 | 0.01866 | 0.17538 | 0.36035 |
| 343 | 0.45744 | -0.03378 | 0.30216 | 0.52504 | 0.6562 | 0.03824 | 0.01894 | 0.17597 | 0.36189 |
| 342 | 0.45925 | -0.03266 | 0.30293 | 0.52598 | 0.6574 | 0.03794 | 0.01852 | 0.17662 | 0.36227 |
| 341 | 0.46058 | -0.03118 | 0.30395 | 0.52785 | 0.65858 | 0.03829 | 0.01857 | 0.1768 | 0.36531 |
| 340 | 0.46229 | -0.03077 | 0.30464 | 0.5293 | 0.66152 | 0.03804 | 0.01897 | 0.17693 | 0.36645 |
| 339 | 0.46384 | -0.03041 | 0.30501 | 0.52961 | 0.66335 | 0.03923 | 0.0198 | 0.17734 | 0.36776 |
| 338 | 0.46541 | -0.02969 | 0.30658 | 0.53075 | 0.66432 | 0.0387 | 0.01887 | 0.17829 | 0.36971 |
| 337 | 0.46686 | -0.02925 | 0.30703 | 0.53318 | 0.66625 | 0.03851 | 0.01898 | 0.17912 | 0.37158 |
| 336 | 0.46897 | -0.02824 | 0.30737 | 0.53452 | 0.66857 | 0.03936 | 0.01909 | 0.17971 | 0.37329 |
| 335 | 0.47098 | -0.02729 | 0.30937 | 0.53619 | 0.6698 | 0.03792 | 0.01879 | 0.18006 | 0.3745 |
| 334 | 0.47174 | -0.02695 | 0.30942 | 0.53849 | 0.67304 | 0.03938 | 0.01961 | 0.18095 | 0.37619 |
| 333 | 0.47427 | -0.02566 | 0.31024 | 0.54153 | 0.67474 | 0.03942 | 0.0193 | 0.18164 | 0.37827 |
| 332 | 0.47619 | -0.02575 | 0.31079 | 0.54271 | 0.67668 | 0.03945 | 0.01947 | 0.18253 | 0.38006 |
| 331 | 0.4772 | -0.02467 | 0.31193 | 0.54364 | 0.67897 | 0.04013 | 0.0201 | 0.18321 | 0.38158 |
| 330 | 0.48123 | -0.02259 | 0.31359 | 0.54626 | 0.68238 | 0.0399 | 0.01957 | 0.184 | 0.38432 |
| 329 | 0.48201 | -0.02224 | 0.31449 | 0.54899 | 0.68458 | 0.04072 | 0.02059 | 0.1846 | 0.38642 |
| 328 | 0.48455 | -0.0205 | 0.31556 | 0.55181 | 0.68658 | 0.0403 | 0.02011 | 0.18578 | 0.38797 |
| 327 | 0.48747 | -0.01951 | 0.31654 | 0.55367 | 0.68845 | 0.04035 | 0.02003 | 0.18609 | 0.39089 |
| 326 | 0.48996 | -0.01886 | 0.31733 | 0.55584 | 0.69258 | 0.04182 | 0.02138 | 0.18699 | 0.39298 |
| 325 | 0.49236 | -0.01715 | 0.31905 | 0.55732 | 0.69472 | 0.04145 | 0.02089 | 0.18885 | 0.39562 |
| 324 | 0.4949 | -0.0164 | 0.32057 | 0.56087 | 0.69755 | 0.04121 | 0.02107 | 0.18935 | 0.3977 |
| 323 | 0.49762 | -0.01533 | 0.32137 | 0.56323 | 0.69988 | 0.04141 | 0.02112 | 0.19072 | 0.39964 |
| 322 | 0.50039 | -0.01436 | 0.3225 | 0.56641 | 0.70354 | 0.04172 | 0.02109 | 0.19158 | 0.40175 |
| 321 | 0.50377 | -0.01332 | 0.32486 | 0.56958 | 0.70619 | 0.04218 | 0.02122 | 0.19305 | 0.40409 |
| 320 | 0.50659 | -0.01089 | 0.32621 | 0.5714 | 0.70881 | 0.04211 | 0.021 | 0.19345 | 0.4062 |
| 319 | 0.50949 | -0.0104 | 0.32729 | 0.57429 | 0.71141 | 0.04195 | 0.02113 | 0.19421 | 0.40954 |
| 318 | 0.51223 | -0.01015 | 0.32864 | 0.57644 | 0.71424 | 0.04259 | 0.02092 | 0.196 | 0.41241 |
| 317 | 0.51638 | -0.00762 | 0.33053 | 0.5802 | 0.71725 | 0.0423 | 0.02156 | 0.19719 | 0.41404 |
| 316 | 0.51926 | -0.00752 | 0.33149 | 0.58338 | 0.72032 | 0.04313 | 0.02197 | 0.19757 | 0.41793 |
| 315 | 0.52226 | -0.00625 | 0.33361 | 0.5871 | 0.72398 | 0.04336 | 0.02174 | 0.19868 | 0.42024 |
| 314 | 0.52596 | -0.00493 | 0.33478 | 0.58943 | 0.72635 | 0.04368 | 0.02179 | 0.20022 | 0.4232 |
| 313 | 0.52879 | -0.00398 | 0.33637 | 0.59332 | 0.73015 | 0.04444 | 0.02251 | 0.20146 | 0.42573 |
| 312 | 0.53238 | -0.00243 | 0.33778 | 0.59606 | 0.73232 | 0.04364 | 0.02229 | 0.20335 | 0.42844 |
| 311 | 0.53708 | -0.00183 | 0.33958 | 0.59881 | 0.73644 | 0.04459 | 0.0224 | 0.20399 | 0.43134 |
| 310 | 0.53949 | 0.000029 | 0.3409 | 0.60247 | 0.73871 | 0.04465 | 0.02321 | 0.20522 | 0.43444 |
| 309 | 0.54436 | 0.00151 | 0.34271 | 0.60484 | 0.74197 | 0.0445 | 0.02281 | 0.20662 | 0.43769 |
| 308 | 0.54039 | -0.0098 | 0.34209 | 0.60878 | 0.74611 | 0.04469 | 0.02279 | 0.20856 | 0.44115 |
| 307 | 0.54377 | -0.0091 | 0.34205 | 0.61305 | 0.74854 | 0.04542 | 0.02328 | 0.20921 | 0.44383 |
| 306 | 0.54687 | -0.0078 | 0.34367 | 0.61509 | 0.75153 | 0.0453 | 0.02291 | 0.21017 | 0.44773 |
| 305 | 0.55139 | -0.00615 | 0.34545 | 0.61968 | 0.75517 | 0.04548 | 0.02321 | 0.21153 | 0.45043 |
| 304 | 0.55589 | -0.00458 | 0.3473 | 0.62227 | 0.75823 | 0.04624 | 0.02376 | 0.21302 | 0.45402 |
| 303 | 0.55925 | -0.00367 | 0.34923 | 0.62597 | 0.76138 | 0.04631 | 0.02374 | 0.21456 | 0.45795 |
| 302 | 0.5648 | -0.00174 | 0.35114 | 0.62918 | 0.76499 | 0.0467 | 0.02403 | 0.21609 | 0.46256 |
| 301 | 0.56898 | -0.000471 | 0.35357 | 0.63359 | 0.76836 | 0.04744 | 0.02482 | 0.21787 | 0.46607 |
| 300 | 0.5731 | 0.000693 | 0.35494 | 0.63713 | 0.77191 | 0.04741 | 0.02442 | 0.2189 | 0.47139 |
| 299 | 0.57882 | 0.00301 | 0.35668 | 0.64024 | 0.775 | 0.04764 | 0.02488 | 0.22067 | 0.47555 |
| 298 | 0.58353 | 0.00385 | 0.35899 | 0.64412 | 0.77871 | 0.04823 | 0.02543 | 0.22226 | 0.48082 |
| 297 | 0.58879 | 0.00605 | 0.36162 | 0.64843 | 0.78234 | 0.0484 | 0.02553 | 0.2231 | 0.48622 |
| 296 | 0.59338 | 0.00793 | 0.36339 | 0.65176 | 0.78676 | 0.04895 | 0.02566 | 0.22471 | 0.49251 |
| 295 | 0.5991 | 0.0098 | 0.36539 | 0.6562 | 0.78945 | 0.04959 | 0.02583 | 0.22688 | 0.50053 |
| 294 | 0.60502 | 0.01213 | 0.36812 | 0.66027 | 0.79285 | 0.04979 | 0.02634 | 0.22789 | 0.5098 |
| 293 | 0.61005 | 0.01364 | 0.37028 | 0.66432 | 0.79585 | 0.05025 | 0.02719 | 0.22986 | 0.52337 |
| 292 | 0.61547 | 0.01469 | 0.37237 | 0.66836 | 0.79984 | 0.05052 | 0.0271 | 0.23174 | 0.5417 |
| 291 | 0.62179 | 0.01641 | 0.37488 | 0.67254 | 0.80371 | 0.05106 | 0.02714 | 0.23347 | 0.56984 |
| 290 | 0.62722 | 0.01858 | 0.37661 | 0.67761 | 0.80778 | 0.05137 | 0.02765 | 0.2358 | 0.61206 |
| 289 | 0.6337 | 0.02107 | 0.37882 | 0.6808 | 0.81117 | 0.05178 | 0.02773 | 0.23845 | 0.6738 |
| 288 | 0.63942 | 0.02205 | 0.38108 | 0.68606 | 0.81491 | 0.05253 | 0.02809 | 0.24193 | 0.76003 |
| 287 | 0.64582 | 0.02429 | 0.38377 | 0.69021 | 0.8184 | 0.05222 | 0.02804 | 0.24549 | 0.87116 |
| 286 | 0.65198 | 0.0251 | 0.38592 | 0.69479 | 0.82246 | 0.05328 | 0.02859 | 0.24901 | 0.9937 |
| 285 | 0.65823 | 0.02776 | 0.38776 | 0.70006 | 0.82714 | 0.05424 | 0.02942 | 0.25374 | 1.10453 |
| 284 | 0.66535 | 0.02827 | 0.39062 | 0.70451 | 0.83025 | 0.05386 | 0.02889 | 0.25881 | 1.18034 |
| 283 | 0.67133 | 0.0297 | 0.39264 | 0.7095 | 0.8343 | 0.05397 | 0.02953 | 0.26256 | 1.22084 |
| 282 | 0.67805 | 0.03105 | 0.39532 | 0.71447 | 0.83881 | 0.05488 | 0.03021 | 0.26508 | 1.24054 |
| 281 | 0.68392 | 0.03159 | 0.39664 | 0.71987 | 0.84295 | 0.05554 | 0.0306 | 0.26804 | 1.26573 |
| 280 | 0.69051 | 0.03311 | 0.39944 | 0.72428 | 0.84681 | 0.05597 | 0.03084 | 0.27063 | 1.30023 |
| 279 | 0.698 | 0.03392 | 0.40157 | 0.72959 | 0.85089 | 0.05691 | 0.03126 | 0.27257 | 1.34533 |
| 278 | 0.7051 | 0.03486 | 0.40343 | 0.7349 | 0.85452 | 0.0569 | 0.03184 | 0.27504 | 1.38739 |
| 277 | 0.7105 | 0.03542 | 0.40572 | 0.74008 | 0.85834 | 0.05747 | 0.03178 | 0.27725 | 1.41047 |
| 276 | 0.71859 | 0.03658 | 0.40767 | 0.74567 | 0.86357 | 0.05821 | 0.03229 | 0.27996 | 1.41758 |
| 275 | 0.72647 | 0.03825 | 0.41064 | 0.75098 | 0.86639 | 0.05863 | 0.03247 | 0.28083 | 1.41016 |
| 274 | 0.73294 | 0.04007 | 0.41294 | 0.75568 | 0.87057 | 0.05886 | 0.03292 | 0.28244 | 1.38911 |
| 273 | 0.74159 | 0.04101 | 0.41543 | 0.76041 | 0.87338 | 0.0595 | 0.03333 | 0.28334 | 1.36266 |
| 272 | 0.74851 | 0.04121 | 0.41721 | 0.7663 | 0.87713 | 0.06004 | 0.03378 | 0.28356 | 1.3328 |
| 271 | 0.75564 | 0.04301 | 0.41988 | 0.76984 | 0.88105 | 0.06105 | 0.0342 | 0.28347 | 1.30145 |
| 270 | 0.76259 | 0.0444 | 0.42184 | 0.77385 | 0.88366 | 0.0611 | 0.03416 | 0.28387 | 1.26936 |
| 269 | 0.7701 | 0.04553 | 0.42361 | 0.7784 | 0.88627 | 0.06197 | 0.0351 | 0.28349 | 1.23585 |
| 268 | 0.77762 | 0.04773 | 0.42612 | 0.78275 | 0.88898 | 0.06217 | 0.03497 | 0.28347 | 1.19845 |
| 267 | 0.78557 | 0.04945 | 0.42877 | 0.78632 | 0.89146 | 0.06256 | 0.03547 | 0.28299 | 1.15969 |
| 266 | 0.79197 | 0.05149 | 0.43053 | 0.78981 | 0.89427 | 0.06357 | 0.03607 | 0.28263 | 1.11849 |
| 265 | 0.79981 | 0.05362 | 0.43237 | 0.79398 | 0.89648 | 0.06379 | 0.03611 | 0.28231 | 1.07809 |
| 264 | 0.80661 | 0.05548 | 0.43539 | 0.79719 | 0.89823 | 0.06381 | 0.0362 | 0.282 | 1.04005 |
| 263 | 0.81275 | 0.05726 | 0.43725 | 0.8006 | 0.90056 | 0.0645 | 0.03706 | 0.28151 | 1.00372 |
| 262 | 0.81983 | 0.05784 | 0.4391 | 0.80331 | 0.90263 | 0.06492 | 0.03732 | 0.28092 | 0.96826 |
| 261 | 0.82489 | 0.05923 | 0.44117 | 0.80684 | 0.90443 | 0.06487 | 0.0371 | 0.28085 | 0.93633 |
| 260 | 0.83152 | 0.06071 | 0.44272 | 0.80858 | 0.90616 | 0.06574 | 0.03781 | 0.28025 | 0.90472 |
| 259 | 0.83598 | 0.06193 | 0.44453 | 0.8106 | 0.90711 | 0.06616 | 0.03816 | 0.27964 | 0.87548 |
| 258 | 0.84173 | 0.06331 | 0.44578 | 0.8136 | 0.90867 | 0.06682 | 0.03824 | 0.27935 | 0.84892 |
| 257 | 0.84647 | 0.06452 | 0.44725 | 0.81602 | 0.90973 | 0.06704 | 0.0381 | 0.27862 | 0.82518 |
| 256 | 0.85003 | 0.06562 | 0.44896 | 0.8177 | 0.91142 | 0.06757 | 0.0389 | 0.27848 | 0.8027 |
| 255 | 0.85455 | 0.06652 | 0.45094 | 0.81946 | 0.91309 | 0.06832 | 0.03934 | 0.27853 | 0.78352 |
| 254 | 0.85889 | 0.06754 | 0.45229 | 0.82074 | 0.91386 | 0.06833 | 0.0394 | 0.27816 | 0.76487 |
| 253 | 0.8624 | 0.06895 | 0.45424 | 0.82313 | 0.91459 | 0.06895 | 0.03967 | 0.27806 | 0.75011 |
| 252 | 0.86563 | 0.06948 | 0.45571 | 0.82477 | 0.91532 | 0.06915 | 0.03963 | 0.27854 | 0.73567 |
| 251 | 0.86948 | 0.07058 | 0.45674 | 0.82578 | 0.9169 | 0.06989 | 0.04038 | 0.27876 | 0.72444 |
| 250 | 0.87183 | 0.07157 | 0.45822 | 0.82797 | 0.91759 | 0.06998 | 0.04034 | 0.27884 | 0.71459 |
| 249 | 0.8754 | 0.07252 | 0.46016 | 0.83015 | 0.91883 | 0.07053 | 0.04105 | 0.27892 | 0.70734 |
| 248 | 0.878 | 0.07355 | 0.46221 | 0.83159 | 0.92024 | 0.07149 | 0.0413 | 0.27951 | 0.70201 |
| 247 | 0.88052 | 0.07431 | 0.4634 | 0.83306 | 0.92141 | 0.07175 | 0.04141 | 0.28038 | 0.69943 |
| 246 | 0.88317 | 0.07482 | 0.46476 | 0.83487 | 0.92235 | 0.07249 | 0.04207 | 0.27989 | 0.69878 |
| 245 | 0.88551 | 0.07601 | 0.46624 | 0.83629 | 0.9238 | 0.07248 | 0.04215 | 0.28105 | 0.70252 |
| 244 | 0.8886 | 0.07683 | 0.46895 | 0.83858 | 0.92522 | 0.07354 | 0.04311 | 0.28229 | 0.71095 |
| 243 | 0.89168 | 0.07772 | 0.47048 | 0.83997 | 0.92658 | 0.07343 | 0.04269 | 0.28286 | 0.72718 |
| 242 | 0.8943 | 0.07811 | 0.47219 | 0.842 | 0.92858 | 0.07458 | 0.04341 | 0.28483 | 0.75265 |
| 241 | 0.89655 | 0.07841 | 0.47382 | 0.84363 | 0.92983 | 0.07582 | 0.04454 | 0.28684 | 0.7942 |
| 240 | 0.90104 | 0.07922 | 0.47602 | 0.84515 | 0.93167 | 0.07675 | 0.04508 | 0.28947 | 0.85883 |
| 239 | 0.90456 | 0.08038 | 0.4782 | 0.84774 | 0.934 | 0.07785 | 0.04621 | 0.29289 | 0.9566 |
| 238 | 0.90729 | 0.0815 | 0.48025 | 0.85041 | 0.93524 | 0.07843 | 0.04662 | 0.29782 | 1.09924 |
| 237 | 0.9116 | 0.08112 | 0.48183 | 0.85221 | 0.93649 | 0.07914 | 0.0473 | 0.30477 | 1.30237 |
| 236 | 0.9166 | 0.08215 | 0.48412 | 0.85477 | 0.93923 | 0.08078 | 0.04808 | 0.31365 | 1.57474 |
| 235 | 0.92116 | 0.08215 | 0.48633 | 0.85799 | 0.94143 | 0.08211 | 0.04953 | 0.32581 | 1.9243 |
| 234 | 0.92534 | 0.08145 | 0.48787 | 0.86036 | 0.94396 | 0.08369 | 0.05057 | 0.33987 | 2.3189 |
| 233 | 0.93186 | 0.08191 | 0.48949 | 0.86323 | 0.9471 | 0.08536 | 0.05189 | 0.35854 | 2.66151 |
| 232 | 0.93826 | 0.08156 | 0.49144 | 0.86696 | 0.95038 | 0.08713 | 0.05323 | 0.38071 | 2.84392 |
| 231 | 0.94624 | 0.08092 | 0.49266 | 0.87029 | 0.9538 | 0.08959 | 0.05476 | 0.40466 | 2.91346 |
| 230 | 0.954 | 0.08068 | 0.49436 | 0.87346 | 0.95721 | 0.09142 | 0.05613 | 0.43014 | 2.9283 |
| 229 | 0.9644 | 0.07875 | 0.49582 | 0.87734 | 0.96167 | 0.09343 | 0.05772 | 0.45615 | 2.94884 |
| 228 | 0.97296 | 0.07762 | 0.49559 | 0.88273 | 0.96588 | 0.09556 | 0.06005 | 0.48231 | 2.95349 |
| 227 | 0.9836 | 0.07544 | 0.49541 | 0.8855 | 0.97085 | 0.09905 | 0.0618 | 0.50968 | 2.95321 |
| 226 | 0.99502 | 0.07219 | 0.49509 | 0.88998 | 0.9778 | 0.10206 | 0.06371 | 0.53898 | 2.95568 |
| 225 | 1.00775 | 0.06821 | 0.49438 | 0.89686 | 0.9841 | 0.10545 | 0.06612 | 0.57055 | 2.95549 |
| 224 | 1.0216 | 0.06298 | 0.49267 | 0.90049 | 0.99207 | 0.10908 | 0.06877 | 0.60503 | 2.94825 |
| 223 | 1.03654 | 0.05827 | 0.49111 | 0.90675 | 1.00071 | 0.11354 | 0.07164 | 0.64398 | 2.94319 |
| 222 | 1.0555 | 0.05196 | 0.48844 | 0.91419 | 1.01143 | 0.11864 | 0.07603 | 0.68828 | 2.94062 |
| 221 | 1.07164 | 0.046 | 0.48489 | 0.92082 | 1.02206 | 0.12441 | 0.0805 | 0.73806 | 2.92207 |
| 220 | 1.09214 | 0.03723 | 0.48019 | 0.93125 | 1.03596 | 0.13067 | 0.08484 | 0.79581 | 2.90803 |
| 219 | 1.11252 | 0.02911 | 0.47415 | 0.93942 | 1.05037 | 0.13806 | 0.08946 | 0.86145 | 2.89455 |
| 218 | 1.13434 | 0.0211 | 0.46844 | 0.9506 | 1.06634 | 0.14524 | 0.0957 | 0.93629 | 2.87492 |
| 217 | 1.15632 | 0.01226 | 0.46231 | 0.95944 | 1.08481 | 0.15456 | 0.10136 | 1.01869 | 2.85698 |
| 216 | 1.1815 | 0.00109 | 0.4535 | 0.97234 | 1.10397 | 0.16232 | 0.10735 | 1.10785 | 2.83614 |
| 215 | 1.20682 | -0.00778 | 0.44607 | 0.98713 | 1.12462 | 0.17312 | 0.11623 | 1.20035 | 2.8109 |
| 214 | 1.23336 | -0.01946 | 0.43921 | 1.00283 | 1.14475 | 0.18323 | 0.12257 | 1.2984 | 2.78176 |
| 213 | 1.25572 | -0.03056 | 0.4306 | 1.01856 | 1.16854 | 0.19378 | 0.13073 | 1.38778 | 2.75548 |
| 212 | 1.28353 | -0.04263 | 0.42152 | 1.03256 | 1.1907 | 0.20519 | 0.13764 | 1.46873 | 2.72683 |
| 211 | 1.30844 | -0.05489 | 0.41278 | 1.04903 | 1.21009 | 0.21417 | 0.14487 | 1.54238 | 2.69182 |
| 210 | 1.33804 | -0.06636 | 0.40421 | 1.0666 | 1.23327 | 0.22438 | 0.15198 | 1.60031 | 2.66066 |
| 209 | 1.35935 | -0.07913 | 0.39534 | 1.08501 | 1.2544 | 0.23446 | 0.15783 | 1.64481 | 2.63106 |
| 208 | 1.38796 | -0.08926 | 0.3878 | 1.10381 | 1.2764 | 0.24507 | 0.16458 | 1.67266 | 2.59741 |
| 207 | 1.41578 | -0.10063 | 0.38039 | 1.12629 | 1.29716 | 0.25465 | 0.17164 | 1.68199 | 2.5647 |
| 206 | 1.43926 | -0.11073 | 0.37274 | 1.1498 | 1.32383 | 0.26666 | 0.17762 | 1.68482 | 2.53679 |
| 205 | 1.47244 | -0.12417 | 0.36534 | 1.17423 | 1.34962 | 0.27903 | 0.18571 | 1.6771 | 2.48633 |
| 204 | 1.50241 | -0.13598 | 0.35786 | 1.20246 | 1.38382 | 0.29145 | 0.19294 | 1.67362 | 2.45592 |
| 203 | 1.52883 | -0.15072 | 0.35004 | 1.23765 | 1.41688 | 0.30594 | 0.20067 | 1.67531 | 2.42496 |
| 202 | 1.56015 | -0.16955 | 0.34046 | 1.27725 | 1.4594 | 0.32303 | 0.21165 | 1.68913 | 2.38022 |
| 201 | 1.5864 | -0.19016 | 0.32955 | 1.31937 | 1.50847 | 0.34379 | 0.22289 | 1.7273 | 2.31152 |
| 200 | 1.62066 | -0.21554 | 0.31375 | 1.37039 | 1.56819 | 0.36749 | 0.23833 | 1.77676 | 2.26926 |
